# Search for New Aggregable Fragments of Human Insulin

**DOI:** 10.3390/molecules24081600

**Published:** 2019-04-23

**Authors:** Monika Swiontek, Justyna Fraczyk, Joanna Wasko, Agata Chaberska, Lukasz Pietrzak, Zbigniew J. Kaminski, Lukasz Szymanski, Slawomir Wiak, Beata Kolesinska

**Affiliations:** 1 Institute of Organic Chemistry, Lodz University of Technology, Zeromskiego 116, 90-924 Lodz, Poland; monika.swiontek@gmail.com (M.S.); justyna.fraczyk@p.lodz.pl (J.F.); joanna.wasko@p.lodz.pl (J.W.); agata.chaberska@p.lodz.pl (A.C.); zbigniew.kaminski@p.lodz.pl (Z.J.K.); 2 Institute of Mechatronics and Information Systems, Faculty of Electrical, Electronic, Computer and Control Engineering, Lodz University of Technology, Stefanowskiego 18/22, 90-924 Lodz, Poland; lukasz.pietrzak@p.lodz.pl (L.P.); lukasz.szymanski@p.lodz.pl (L.S.); slawomir.wiak@p.lodz.pl (S.W.)

**Keywords:** Aggregation, amyloid deposits, amyloid-like fiber formation, diabetes, SPPS, triazine coupling reagents

## Abstract

In this study, three independent methods were used to identify short fragment of both chains of human insulin which are prone for aggregation. In addition, circular dichroism (CD) research was conducted to understand the progress of aggregation over time. The insulin fragments (deca- and pepta-peptides) were obtained by solid-phase synthesis using 4-(4,6-dimethoxy-1,3,5-triazin-2-yl)-4-methylmorpholinium toluene-4-sulfonate (DMT/NMM/TosO^-^) as a coupling reagent. Systematic studies allowed identification of the new fragments, expected to be engaged in triggering aggregation of the entire structure of human insulin under physiological conditions. It was found that the aggregation process occurs through various structural conformers and may favor the formation of a fibrous structure of aggregate.

## 1. Introduction

Insulin is involved in many bodily processes. It participates in DNA replication and protein synthesis by regulating amino acid uptake and modulating the activity of many enzymes, such as hexokinase, phosphofructokinase, glycogen synthase and glycogen phosphorylase [1]. Insulin is also involved in the biosynthesis of glycogen and its storage in the liver, resulting in lowering of blood glucose. It accelerates the biosynthesis of fats and slows down proteolysis, lipolysis and glucogenesis. It prevents glucose synthesis from non-sugar substrates. Impairment of its overriding functions, through its destruction by autoimmune β cells or loss of biological activity, causes hyperglycemia and is correlated with the development of type I and type II diabetes [2,3]. Human insulin, with a molecular weight of 5808 Da, is formed from 51 amino acid residues. It is built from two chains: Chain A (alpha), containing 21 amino acid residues and chain B (beta), composed of 30 amino acid residues. Both chains are connected by two disulfide bridges formed between cysteines A7–B7 and A20–B19. In chain A there is a third disulfide bridge connecting the two cysteines in position A6–A11. Insulin aggregation is a crucial factor in the development of diabetes [4,5,6,7]. Aggregation of insulin in solution is also an important technological problem for the production, storage and use of insulin preparations [8,9,10]. 

The ability of a protein to aggregate depends on its primary structure. The most important factors contributing to aggregation are the hydrophobicity of amino acid residues, the nature of the amino acid side chains, the balance between polar and non-polar amino acids and the presence of aromatic ring interactions. Many differently structured proteins and polypeptides are affected by inappropriate folding, resulting in the formation of stable and insoluble peptide/protein structures. Altered spatial structures result in the formation of amyloid deposits, leading directly to the development of conformational diseases or amyloidosis [1,2]. In the case of insulin, it is postulated that the formation of amyloid fibers consisting of characteristic structured β-sheets occurs under a wide range of environmental conditions and is accelerated by high temperature and low pH [3,11]. A characteristic feature of amyloidogenic proteins is the presence in their structures of short fragments responsible for initiating aggregation, which are also involved in the stabilization of amyloid fibrils. These fragments, called hot spots, form amyloidogenic cores and can contain up to three or four amino acid residues. In vitro, these undergo aggregation, leading to amyloid-like structures [10,12,13,14,15,16,17]. 

The first atomic-level view of the interactions between segments of insulin, which may form part of a fibrillar spine (“amyloidogenic core”), revealed single crystal structures in the fibril-forming peptide segments A13–A19 H–LYQLENY–OH, A13–A18 H–LYQLEN–OH and B12–B17 H–VEALYL–OH. Biophysical studies suggest that the B chain of insulin, or a segment of it, may be the preliminary determinant of insulin fibrillation. The amyloidogenic core of insulin consists of two antiparallel arranged B12–B17 fragments, which due to their specific mutual orientation form a “steric zip”, which is stabilized by hydrophobic interactions between aliphatic amino acid residues, hydrogen bonds between insulin chains and particularly by the interaction of hydroxyl groups in the tyrosine–tyrosine side chains. This structure interacts with the A13–A19 LYQLENY fragment of chain A, which is involved in the stabilization of the β-structure of the amyloidogenic core [18,19]. At the periphery of the insulin fibrillar spine model, the N- and C-termini retain the native-like structure of the insulin molecule. A similar model, with a “steric zipper” spine and native-like structure on the periphery, was proposed for the designed amyloid of ribonuclease A [20]. These findings were confirmed by Eisenberg, who classified the fibrillary spine of insulin within the face-to-back class of “steric zippers” [19] formed by B12–17 VEALYL, B11–16 LVEALY and A13–18 LYQLEN. These short peptides have been described and characterized by Congo Red (CR), Thioflavin T (ThT), electron microscopy and X-ray diffraction assays. In the literature there is a plenty of examples of peptides as short as three amino acid residues, which form fibrils and which then play a role in aggregation of the whole protein [10,12,13,14,15,16,17]. Results of this studies has been used by Masunov et al. for Molecular Dynamics (MD) studies on full-length insulin amyloid oligomer models [21]. More recently another group also performed MD simulation. Studies of molecular dynamics indicated strong interaction between the docked fragment LVEALYL and fragment B22–B27 RGFFYT. In the best model, RGFFYT was bound to insulin by one hydrogen bond with Phe–B25. It has also been found that the binding of LVEALYL to RGFFYT is driven by electrostatic interaction, because both the ligand and receptor expose charged amino acid residues. Using MD simulations and the Zyggregator method to calculate the propensity profile, it has been predicted that RGFFYT can also self-assemble. It was postulated that presence of VE (fragment 2–3) CS (fragment (11–12) from chain A and FVNQH (fragment 1–5) and RGFF (fragment 22–25) is conducive to the increase of β-strand content [22]. This data were assigned to insulin, but also to a mixture of insulin and fragment LVEALYL (hot spot of insulin). Authors have indicated especially on fragment B22–27 RGFFYT, which interacts with B12–17 LVEALYL and hypothesized that this fragment is prone to aggregation, which when coming back to experimental data was quite surprising, since neither this region nor even neighboring ones were described as amyloidogenic. 

This was our inspiration to check this hypothesis by experiments under physiological conditions (pH = 7.2, 37 °C). Therefore, the main aim of the present study was to conduct a systematic search for new insulin fragments prone to aggregation. Finding aggregating fragments should assist the rational design of hormone aggregation inhibitors, facilitating the search for new drugs for the treatment of diabetes. Short fragments covering the complete structure of human insulin were synthesized and their propensity to aggregate studied, in order to select new aggregating peptides than those previously identified. The sequences of all the fragments of insulin designed for testing are presented in Table 1.

As a positive control in the studies, we used fragments of insulin already described in the literature as having the ability to form aggregates: A13–A19 (H–LYQLENY–OH) (**16**) and B12–B17 (H–VEALYL–OH) (**17**). Fragment B22–B27 (H–RGFFYT–OH) (**18**), which is suspected of having aggregable properties, was also used [22]. 

## 2. Results and Discussion

Peptides **1**–**18** were synthesized according to the solid phase peptide synthesis (SPPS) method using 4-(4,6-dimethoxy-1,3,5-triazin-2-yl)-4-methylmorpholinium toluene-4-sulfonate (DMT/NMM/TosO^-^) as a coupling reagent [23]. The purity of the crude peptides increased from 72–97% to 96–99% after purification by HPLC. The starting point for the studies was the aggregation of already known insulin-inscribed amyloidogenic cores. Two known aggregating fragments derived from A and B chains A13–A19 (H–LYQLENY–OH) (**16**) and B12–B17 (H–VEALYL–OH) (**17**) were used. Also, fragment B22–B27 (H–RGFFYT–OH) (**18**), which is suspected of having this property, was investigated. In addition, human insulin was used in our studies as a reference point. The aggregation process was carried out under physiological conditions: pH, 7.2; temperature, 37 °C. Such mild conditions were selected to mimic, as far as possible, the natural conditions inside the human body. 

**Figure 1:**
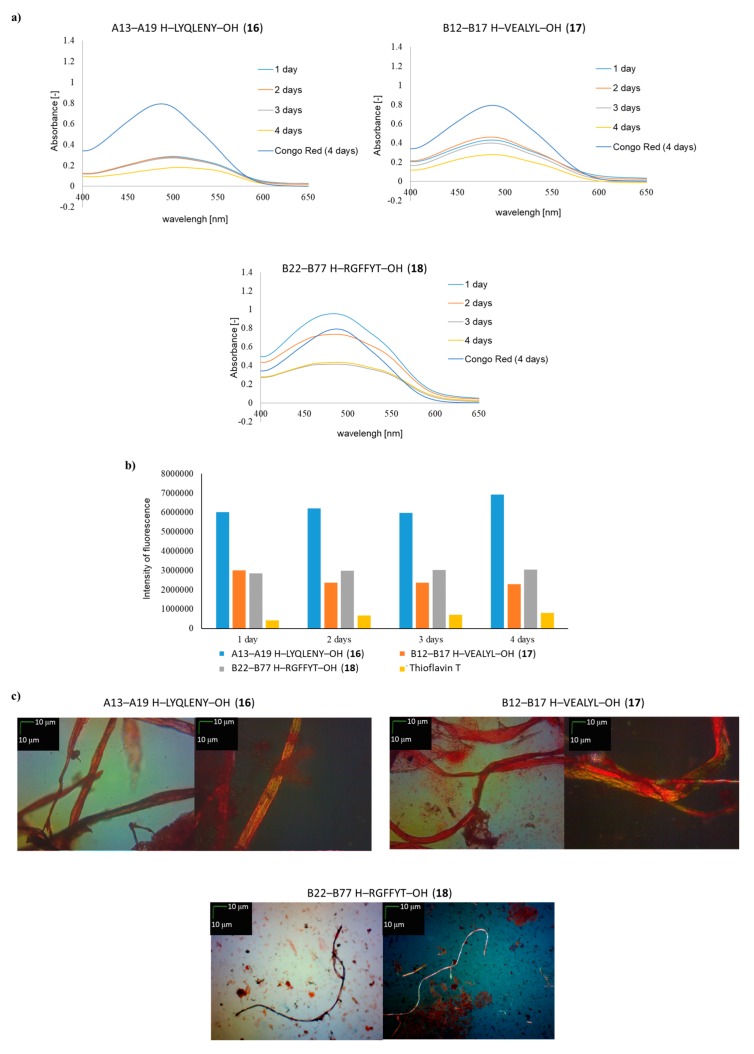

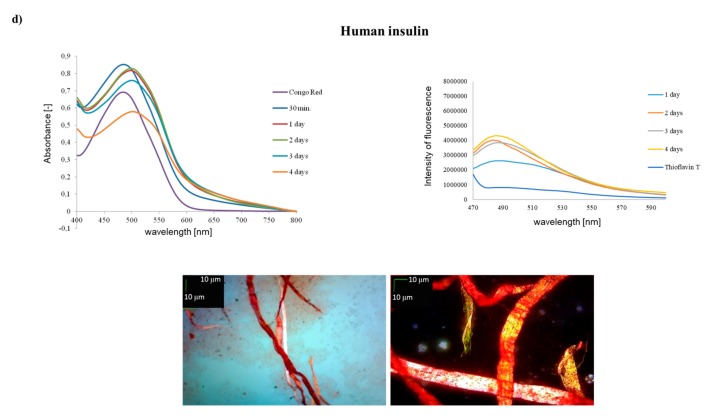
(**a**) UV spectra of A13–A19 H–LYQLENY–OH) (**16**), B12–B17 H–VEALYL–OH (**17**) and B22–B27 H–RGFFYT–OH (**18**) in the presence of Congo Red (CR). Spectra show results obtained on the 1st, 2nd, 3rd and 4th days of incubation; (**b**) fluorescence intensity spectra of peptides **16**, **17** and **18** in the presence of Thioflavin T (ThT), wavelength = 485 nm, 4th day of incubation; (**c**) pictures of **16**, **17** and **18**, without polarized filter (left side), with polarized filter (right side). Scale bars, 10 µm. In all cases, samples were taken for microscopic examination on the 4th day of incubation; (**d**) UV spectra of human insulin in the presence of CR (spectra registered on the 1st, 2nd, 3rd and 4th days of incubation), fluorescence intensity spectra of human insulin in the presence of ThT (spectra registered on the 1st, 2nd, 3rd and 4th days of incubation) and pictures of human insulin without polarized filter (left side), with polarized filter (right side).

The result of the aggregation process is the formation of spatially ordered peptide structures driven by weak interactions between amino-acid residues. It was therefore decided that susceptibility to aggregation would be examined by three independent methods, nonspecific for amyloids, but recommended [24,25,26,27,28,29,30,31,32] for monitoring protein/peptide aggregation. The following tests were used: The Congo Red test, the Thioflavin T assay, and microscopic examination of samples stained with Congo Red. At this stage of the research, using three independent, nonspecific assays did not allow outright differentiation of amyloid fiber from aggregates, but we expected that it would facilitate selection of fragments that are susceptible to aggregation. Based on these independent methods, recommended [24,25,26,27,28,29,30,31,32] for monitoring protein/peptide aggregation, it was found that both B12–B17 and A13–A19 fragments had attributes characteristic of aggregable peptides under physiological conditions. For both insulin aggregating fragments (fragments B12–B17 and A13–A19) known from the literature [18,19], a characteristic decrease in absorbance was observed over time, with a simultaneous shift in the maximum absorbance from 489 nm to 542.5 nm (Figure 1a). In the case of the fragment suspected of aggregation ability, B22–B27, on the first day of aggregation the Congo Red (CR) test showed an increase in absorbance compared to CR with the simultaneous appearance of a new absorbance maximum at 542.5 nm. Measurements on subsequent days of incubation showed a decrease in absorbance, while maintaining the characteristic shift of the maximum absorbance to around 550 nm.

The CR test thus confirmed that the fragment B22–B27 suspected of aggregation properties could be another aggregable human insulin core. The fluorescence assay with Thioflavin T (ThT) also confirmed the aggregable properties of B12–B17 and A13–A19 insulin fragments. On the spectra, a characteristic increase in fluorescence intensity was observed from 7.8 × 10^5^ (for pure incubated Thioflavin T) to 6.9 × 10^6^ for H–LYQLENY–OH (Figure 1b). However, when the increases in the intensity of fluorescence from insulin fragments A13–A19 and B12–B17 were compared, it was noticed that fluorescence intensity increased from 5.6 × 10^6^ to 6.9 × 10^6^ for H–LYQLENY–OH and from 2.8 × 10^6^ to 3 × 10^6^ for H–VEALYL–OH. It is surprising that the increase in fluorescence intensity was lower for the B12–B17 fragment, with documented aggregating properties, than in the case of the B22–B27 fragment, which is only suspected of only having the ability to form aggregates. The results of tests with human insulin indicate that applied, nonspecific tests can be used to search for new fragments with aggregation properties (Figure 1d). In the microscope test, characteristic fibrous structures were observed in both polarized and non-polarized light. The result of the UV assay with CR was also unequivocal. On the UV spectra the characteristic shift of the maximum absorbance and the decrease in absorbance during incubation was observed. Surprising was the result of the ThT assay, where the observed fluorescence intensity value 4.85 × 10^6^ was lower than the data obtained for A13–A19 H–LYQLENY–OH) (**16**). However, the positive result of two of the three independent, nonspecific tests allows the confirmation of aggregation properties of human insulin. Thus, the use of nonspecific methods to study the known aggregable insulin fragments and those suspected of this property, as well as human insulin, has let us use the results of these tests as a benchmark in testing new fragments of insulin, to select regions of the hormone prone to aggregation, which may be the basis for further studies used specifically for amyloid tests.

**Figure 2:**
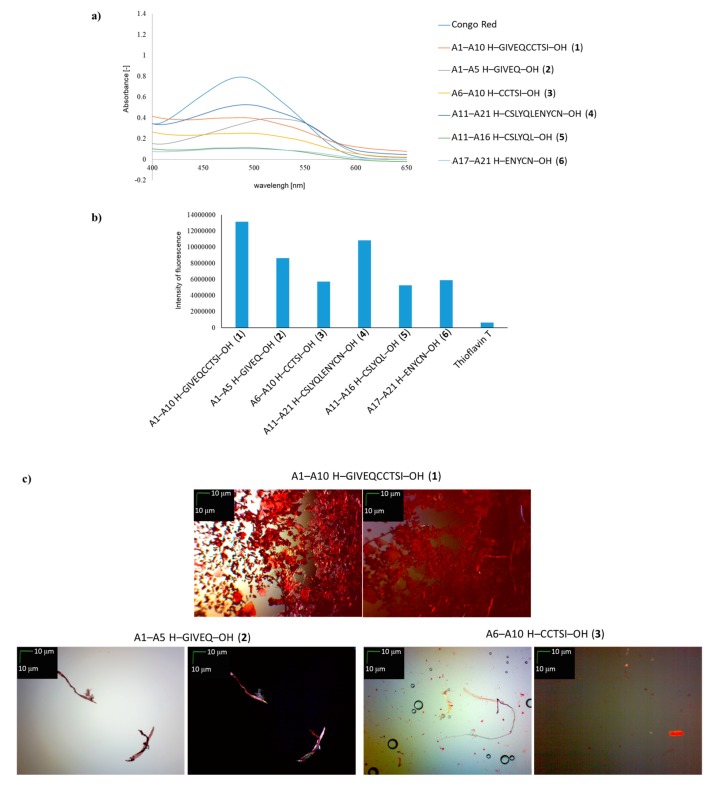

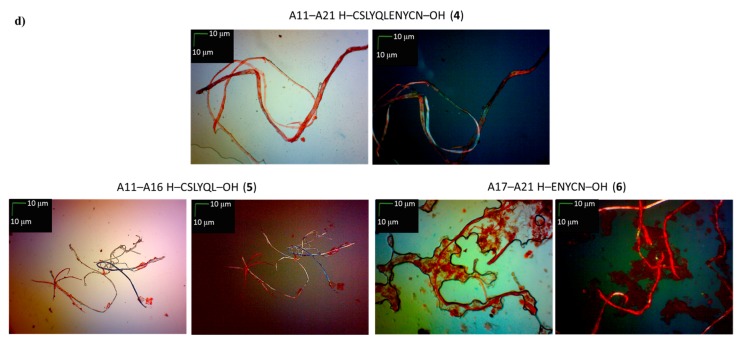
(**a**) UV spectra of H–GIVEQCCTSI–OH (**1**), H–GIVEQ–OH (**2**), H–CCTSI–OH (**3**), H–CSLYQLENYCN–OH (**4**), H–CSLYQL–OH (**5**) and H–ENYCN–OH (**6**) in the presence of CR. Spectra were obtained on the 4th day of incubation; (**b**) fluorescence intensity of **1**–**6** peptides in the presence of ThT, wavelength = 485 nm, 4th day of incubation; (**c**) pictures of **1**–**3** peptides; (**d**) pictures of **4**–**6**, without polarized filter (left side), with polarized filter (right side). Scale bars, 10 µm. In all cases, samples were taken for microscopic examination on the 4th day of incubation.

In the next stage of the study, the same tests were carried out to examine the susceptibility to aggregation of **1**–**6** peptides, which are fragments of the A-chain of insulin (Figure 2). Based on the results of the CR test, all peptides **1**–**6** lowered absorbance in the presence of the dye. The most spectacular decrease in absorbance was observed in the cases of peptides **5** and **6**. All the fragments derived from human insulin chain A showed a characteristic shift of maximum absorption, from 489 nm to 545 nm (Figure 2a). Analysis of the results of the ThT test also confirms the ability of peptides **1**–**6** to aggregate and form aggregates and fibrous structures. In each case, a significant increase in the fluorescence intensity of the dye was observed in the presence of the tested peptides compared to the control, for which Thioflavin T alone was used (Figure 2b). The fluorescence intensity values for deca-peptides **1** and **4** were higher than for the shorter fragments **2**,**3** and **5**,**6** were lower. These results are extremely interesting, particularly as regards peptide **1**, because microscopic studies of this peptide indicated the presence of mainly amorphous structures, with a small proportion of fibrous structures (Figure 2c).

Microscopic studies of the remaining fragments derived from the A-chain of human insulin revealed that characteristic fibrous structures had formed during aggregation in the presence of CR. Fibrous structures were visible both with and without a polarizing filter (Figure 2c,d).

**Figure 3:**
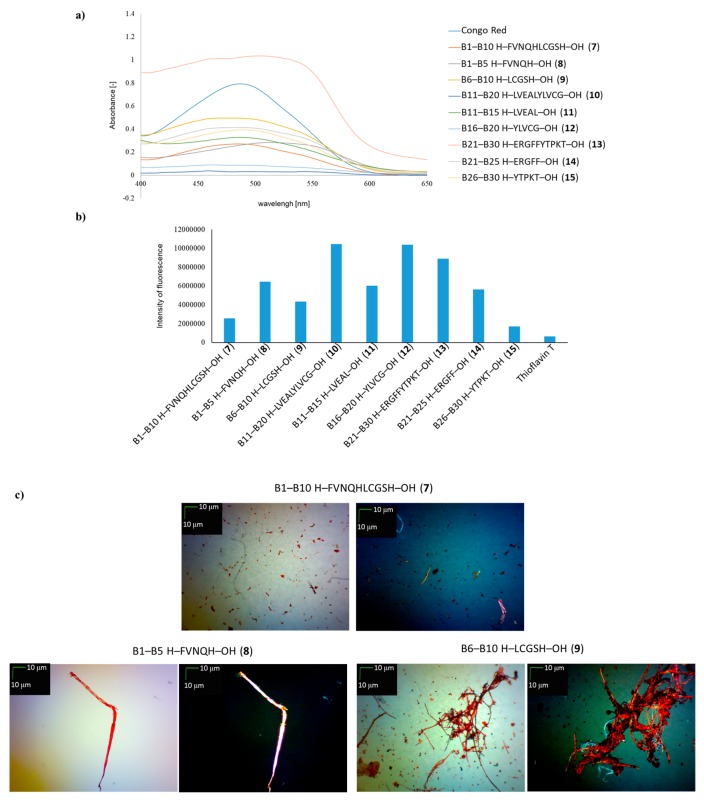

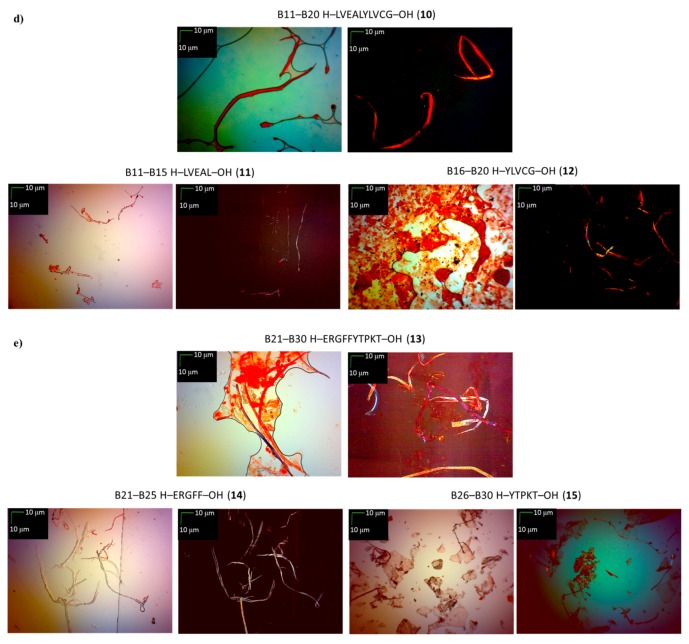
(**a**) UV spectra of H–FVNQHLCGSH–OH (**7**), H–FVNQH–OH (**8**), H–LCGSH–OH (**9**), H–LVEALYLVCG–OH (**10**), H–LVEAL–OH (**11**), H–YLVCG–OH (**12**), H–ERGFFYTPKT–OH (**13**), H–ERGFF--OH (**14**) and H–YTPKT–OH (**15**) in the presence of CR. Spectra were obtained on the 4th day of incubation; (**b**) fluorescence intensity of **7**–**15** peptides in the presence of ThT, wavelength = 485 nm, 4th day of incubation; (**c**) pictures of **7**–**9** peptides; (**d**) pictures of **10**–**12** peptides; (**e**) pictures of **13**–**15** peptides, without polarized filter (left side), with polarized filter (right side). Scale bars, 10 µm. In all cases, samples were taken for microscopic examination on the 4th day of incubation.

In the next stage of the research, the same tests were made on the ability of large and small fragments derived from the B chain of human insulin (peptides **7**–**15**) to form aggregates containing fibrous structures (Figure 3). The CR test showed that, for most fragments of the B-chain of human insulin, characteristic decreases in absorbance occurred and the maximum absorption shifted from 489 nm to 545 nm (Figure 3a), indicating the susceptibility of these fragments to aggregation. Only for peptide **13** was a much higher absorbance compared to CR observed accompanied by the maximum absorbance shift almost to 550 nm. Moreover, the shape of the UV–Vis spectra of peptides **10** and **12** was quite unexpected, because in both cases the spectra were almost flat. Thus, the nonspecific Congo Red assay does not definitely answer which fragments of B chain of insulin are aggregable ones. 

The ThT assay showed for all fragments of B chain (peptides **7**–**15**) a higher fluorescence intensity than the control (Figure 3b). The highest intensity of fluorescence was observed for deca-peptides **10** and **12** as well as for penta-peptide **13**. High fluorescence intensity indicates the ability to form aggregates. This result is also consistent with the data obtained from microscopic examination. The peptides **10**, **12** and **13** formed fibrous structures visible both with and without a polarizing filter (Figure 3d,e). The lowest fluorescence intensity was seen for peptides **7** and **15**, suggesting the lowest potential for aggregation. This result is consistent with the data from microscopic studies, in which mainly amorphous structures were observed, contaminated only with fibrous structures. The results presented in Figure 2 and Figure 3 confirm that, with structures formed as a result of weak interactions, the use of a single analytical method may lead to erroneous conclusions. A summary of the results of CR, ThT and microscopic tests for known aggregable fragments of insulin and tested peptides **1**–**15** is presented in Table 2. 

The assay results indicate that among the tested peptides **1**–**15**, it was not possible to indicate a fragment that in all three tests would give a very good result as in the case of peptide A13–A19 H–LYQLENY–OH (**16**). It was assumed that the positive result of the two independent tests predisposed the examined fragments of insulin to be classified into a group conducive to the aggregation of the hormone.

**Figure 4:**
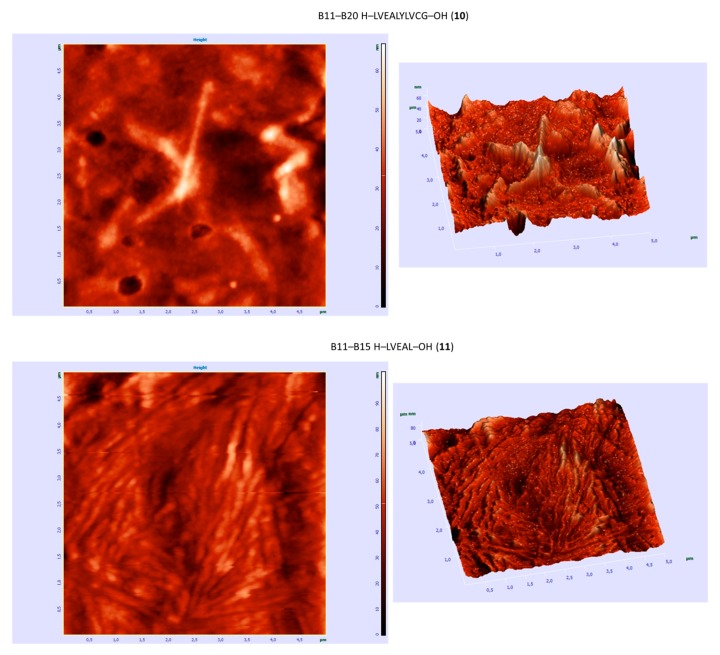

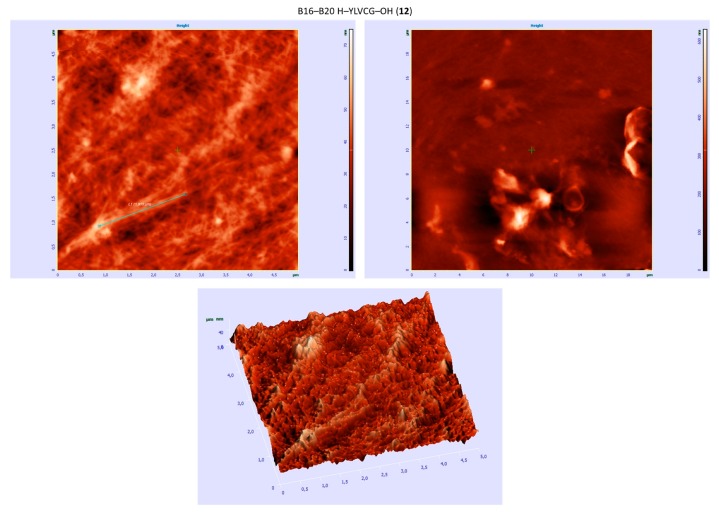
Three-dimensional structures of selected peptides, atomic force microscopy images.

Selected peptides **10**, **11** and **12**, were further examined using an atomic force microscopy (AFM) microscope. The presence of fibrous structures was observed with a tendency to form larger agglomerates (Figure 4).

Research using the AFM technique confirmed the fact that structures in the form of fibrils aggregated with a dominant amorphous phase, which was clearly visible in the case of the peptide **10**. The presence of fibrillar structures were observed in sample **12**, as a major agglomeration of particles. In the case of peptide **11**, a lamellar structure characteristic of the crystalline phase of partially crystalline polymers was seen, presumably indicating the possibility of homogeneous crystallization. However, spherulites were not clearly defined. Weak interactions were most likely responsible for form of organization.

Having demonstrated the ability of several A and B chain fragments to form fibrous structures, studies were continued to analyze the secondary spatial structures formed by fragments of insulin incubated in phosphate buffer solution, pH 7.2, at 0.1 mg/mL concentration at room temperature. For this purpose, selected fragments of human insulin were tested using circular dichroism (CD) spectroscopy. 

Interpretation of the CD spectra was challenging. The general rule states that peptides with a random coil structure have a minimum at 195–200 nm. Peptides rich in β-structures have a minimum at 216 nm. Above that, the progress of aggregation process is accompanied by a shift of the minima to longer wavelengths. This interpretation has been used previously for β-amyloid [33,34,35]. All such shifts refer to situations in which random coil conformation changed via the α-helix, which only in subsequent steps rearranged to amyloid fibrils. Dunstan et al. also describe protofilaments in the preliminary phase of the aggregation of amorphous aggregates as initially containing mainly α structures [36], thus the α-helix structure is postulated on the way/pathway to mature amyloid fibrils. This is what we too have concluded from our research [16].

Due to the poor predictability of this multistage conformational transformation, it was essential to combine different analytic methods to determine whether we were dealing with amorphous aggregates, protofibrils or with mature fibrils. Only mature fibrils contain only β-sheet structures. Moreover, interpretation of CD spectra can be unreliable, especially when it comes to β-structures, which are very often mistaken for α-helix rich structures. There is also a point of structural diversity, which is assigned to β-structures. This diversity refers to the orientation of the peptide backbone and the direction of its twist. Both effects have an influence on the observed spectra. The following configurations should be mentioned: Anti-1 left-twisted β-strand (left-hand twisted antiparallel β-sheet), anti-2 relaxed β-strand (relaxed, slightly right-hand twisted antiparallel β-sheet), anti-3 right-twisted β-strand (right-hand twisted antiparallel β-sheet) and parallel β-strand (parallel β-sheet). All of these are very similar in shape to the distorted α-helix and pose similar minima at 195–200 nm [37,38]. For instance, the anti-1 structure has two characteristic minima at 190–195 nm and 225–230 nm. The anti-2 structure gives one minimum at 180–190 nm and two small minima at 215–220 nm and 235–240 nm, at the very end of the spectrum. Anti-3 also possesses a minimum in the range of 190–200 nm, but also two small maxima in the ranges of 205–210 and 230–235. Finally, the parallel β-strand has one maximum at 200–210 nm and one small minimum at 210–220 nm.

**Figure 5:**
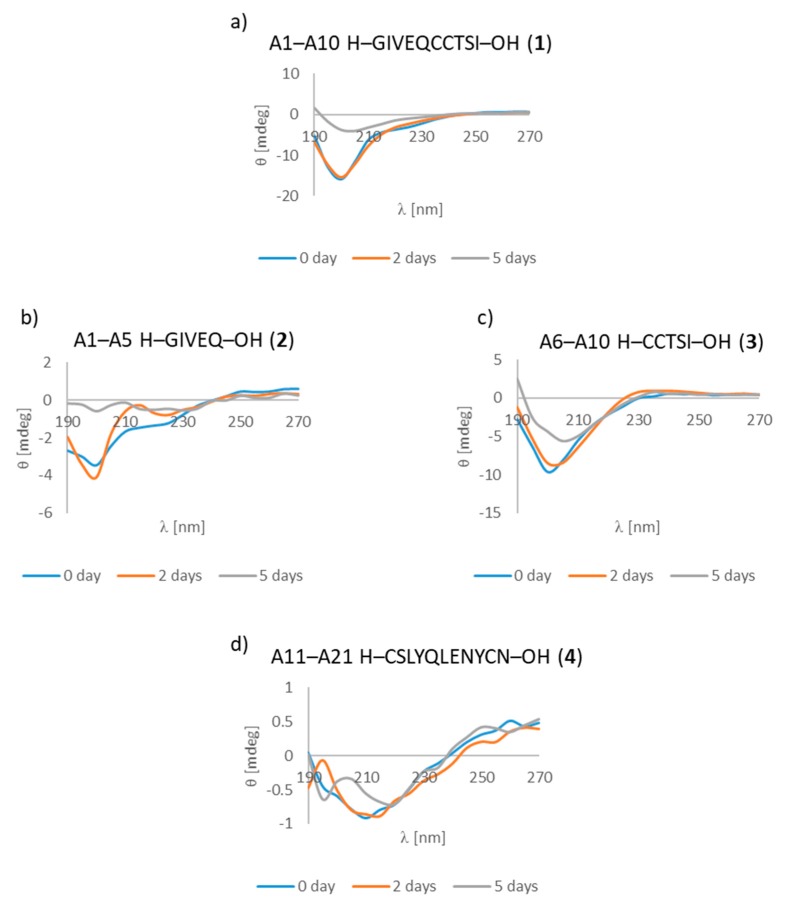
Circular dichroism (CD) spectra of fragments (**a**) A1–A10 (peptide **1**), (**b**) A1–A5 (peptide **2**), (**c**) A6–A10 (peptide **3**), (**d**) A11–A21 (peptide **4**). The peptides were incubated in phosphate buffer solution, pH 7.2, concentration 0.1 mg/mL at room temperature: 0 day (CD spectrum of peptides after dissolving the sample); 2 days (CD spectrum of peptides after 2 days of incubation); 5 days (CD spectrum of peptides after 5 days of incubation).

We first looked for characteristic changes on the CD spectra of the insulin A chain. A characteristic shift of the minimum from 195–200 nm to 205 nm after five days of incubation was observed for deca-peptide **1** (A1–A10 fragment). This effect could indicate structural changes in the peptide, starting from a random coil structure to a more β-rich structure. These results are supported by our microscopic measurements, in which clusters of mainly fibrous and amorphous structures were observed. The same effect was found for penta-peptide **3** (A6–A10 fragment). This shift of the minimum from 195 nm to 215 nm could indicate an anti-3 right twisted β-strand. It is also worth mentioning that microscopic studies also revealed fibrous structures for this peptide. For penta-peptide **2** (fragment A1–A5), changes were noted in the spectra. After two days of incubation there was a random coil structure, but after three more days of incubation the CD spectrum began changing. The shape of spectra became irregular and it was not easy to assign a definite conformation (Figure 5a–c). Nevertheless, a fibrous structure was observed under the microscope. Very interesting spectra were also recorded for deca-peptide **4** (A11–A21 fragment), strongly suggesting the presence of structure β, which could be a mixture of anti-1 and anti-3 structures (Figure 5d). It is worth emphasizing that this peptide contains the shorter A13–A19 fragment, which has been described in the literature as an amyloidogenic core of insulin. Again, fibers were seen under the microscope. 

**Figure 6:**
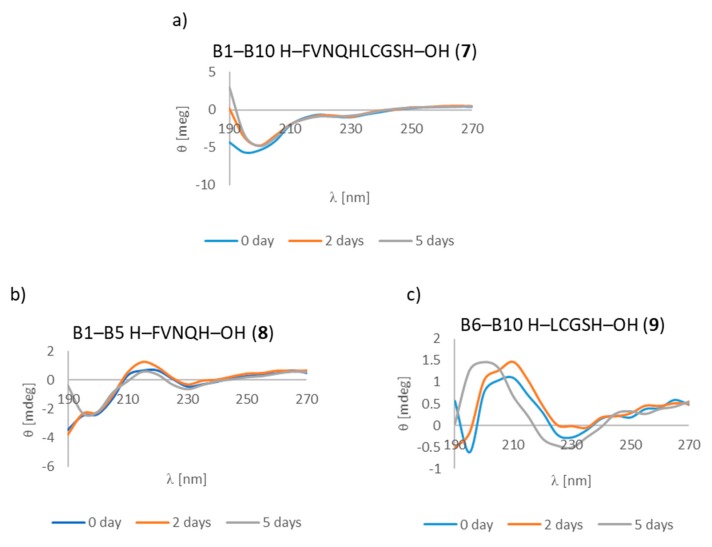
CD spectra of fragments (**a**) B1–B10 (peptide **7**), (**b**) B1–B5 (peptide **8**), (**c**) B6–B10 (peptide **9**). Experimental conditions were identical to those described above.

Turning to chain B of insulin, we observed changes in some regions of the spectra, which could indicate the presence of structure β. These were visible in three fragments. Deca-peptide **7** (B1–B10 fragment) had a minimum at 195 nm from the very beginning of incubation. However, between the second and fifth days this minimum shifted to 205 nm and was accompanied by the appearance of a minimum at 221 nm, which could indicate structure β. Microscope measurements again revealed aggregates. Both penta-peptides **8** and **9** derived from deca-peptide **7** (B1–B10 fragment) had an aggregating propensity. In the case of peptide **8** (B1–B5 fragment), both anti-1 and anti-3 structures were observed, and for fragment B6–B10 (peptide **9**) anti-1 and anti-3 structures slowly changed to relaxed β-strands (Figure 6a–c). Surprisingly, these had never been considered as fragments that undergo aggregation processes. 

**Figure 7:**
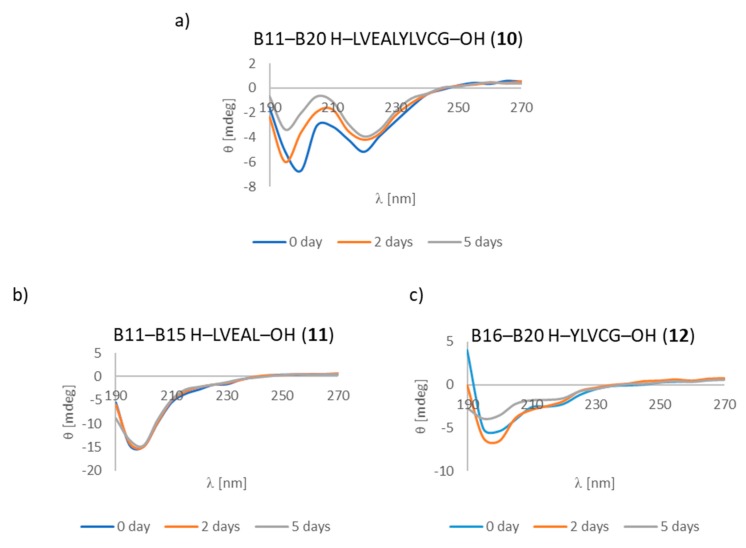
CD spectra of fragments (**a**) B11–B20 (peptide **10**), (**b**) B11–B15 (peptide **11**), (**c**) B16–B20 (peptide **12**). Experimental conditions were identical to those described above.

Very interesting changes in the CD spectra were observed for fragments belonging to deca-peptide **10** (B11–B20 fragment). Deca-peptide **10** has a minima characteristic for an anti-1 structure, but the shorter B11–B15 fragment belonging to deca-peptide **10** only showed a random coil structure. It is also very interesting to note that fragment B16–B20 (peptide **12**) (Figure 7a–c) behaved in the same way as fragment B1–B10 (peptide **7**), described previously. 

These results suggest that the amino acid tyrosine could have an essential role in the aggregation of fragment B16–B20 (peptide **12**). Fragment B12–B17 likewise appears to play an important role in the aggregation of insulin, as part of a “steric zipper” [19]. 

Considering molecular dynamics studies [22], which have suggested, that there may be more short fragments and even amino acid residues affecting the increase of content of β-strand, the results of our studies can support this hypothesis. We found, that fragments A11–21, A11–16 undergo aggregation, and they cover the A11–A12 fragment and the A15 residue which are indicated as crucial for aggregation. Found by us, fragments A1–A10, A1–A5 possess aggregation propensity and both contain the A3 and A4 residues also influencing aggregation. We have also confirmed, that fragment B22–B27, and also a shorter one, B21–B25 undergo aggregation processes. These results are not contradicting with MD simulations results. Moreover, fragment B1–5 with a β-strand conformation assigned by MD simulations, also showed aggregating propensity according to our experimental data. 

## 3. Materials and Methods 

Solid phase peptide synthesis (SPPS) was used to synthesize peptides **1**–**18** according to the Fmoc methodology. Fmoc-protected amino acids were purchased from Novabiochem (San Diego, CA, USA) or Bachem AG (Bubendorf, Switzerland). Human insulin was purchased from Sigma-Aldrich (Saint Louis, MO, USA). 

### 3.1. Synthesis of Peptides

#### 3.1.1. Loading of the 2-Chlorotrityl Chloride Resin (GP1) 

The amino acid (3 equivalents relative to the resin) and EtN^i^Pr_2_ (6 equivalents relative to the resin) were dissolved in CH_2_Cl_2_ (10 mL/g resin), containing if necessary, a small amount of *N*,*N*-dimethylformamide (DMF) to facilitate dissolution of the amino acid. The 2-chlorotrityl chloride resin was preswollen in CH_2_Cl_2_ for 1 h, and then the solution containing amino acid was added. The mixture was shaken for 30–120 min, then the resin was washed with CH_2_Cl_2_/MeOH/EtN_i_Pr_2_ 17:2:1 (3×), DMF (2×) and CH_2_Cl_2_ (3×). The functionalized resin was dried in a vacuum desiccator to constant mass. Deprotection of the first amino acid was prolongated (2 × 30 min).

#### 3.1.2. Standard Coupling Procedure (GP 2) 

Three equivalents of amino acid and 6 equivalents of *N*-methylmorpholine (NMM) were mixed and added to the resin, followed by the addition of 3 equivalents of coupling reagent (DMT/NMM/TosO^-^). The solution was added to the resin and shaken for 1–2 h. The completion of the reaction was monitored using the Kaiser test. 

#### 3.1.3. Fmoc- Deprotection (GP 3) 

The Fmoc protecting group was removed by placing the resin in a solution of 25% piperidine in with DMF for 30 min. The completion of the reaction was monitored using the Kaiser test.

#### 3.1.4. Cleavage from the Resin (GP 4) 

Peptides (**1**–**18**) were cleaved from the resin using a mixture of 95% TFA (2,2,2–trifluoroacetic acid)/2.5% H_2_O/2.5% TIS (triisopropylsilane) (ca. 2 mL/0.1 g resin). Cleavage was performing over 3 h, then the resin was filtered off and the filtrate was evaporated. The residue was treated with Et_2_O to precipitate the peptide. The resulting solid was filtered off and washed with Et_2_O. The crude product was lyophilized and identified by MS. Its purity was determined by RP-HPLC. All HPLC spectra and MS spectra are presented in Supplementary Materials).

### 3.2. Peptides ***1***–***18*** Analysis

Analytical RP-HPLC. Performed on a Waters HPLC system (Waters Corporation, Milford, MA, USA), using a Kinetex Reversed Phase C18 column (100 × 4.6 mm). A gradient of 0.1% TFA in H_2_O (B) and 0.1% TFA in CH_3_CN (A), at a flow rate 0.4 mL/min was used with UV detection at 220 and 254 nm. All HPLC spectra of crude **1**–**18** peptides are available in Supplementary Materials: Figures S1, S3, S5, S7, S9, S11, S13, S15, S17, S19, S21, S23, S25, S27, S29, S31, S33 and S35.

MS analysis. Performed on MS Bruker microOTOF-QIII (Bruker Corporation, Billerica, MA, USA). MS spectra of **1**–**18** peptides are available in Supplementary Materials: Figures S2, S4, S6, S8, S10, S12, S14, S16, S18, S20, S22, S24, S26, S28, S30, S32, S34 and S36. 

Preparative HPLC. Performed on a CombiFlash, EZPrep, Teledyne ISCO (Lincoln, Nebraska, USA) using a Supelco Discovery BIO Wide Pore C18 column (25 cm × 21.2 mm, 10 mm; Sigma-Aldrich); flow rate, 5 mL/min; detection wavelengths, 220 and 254 nm) with gradient ratio A (0.1% TFA in MeCN) and B (0.1% TFA in H_2_O) 0:100 to 18:82 in 30 min, followed by an isocratic run for 5 min.

Spectroscopic measurements. Performed on UV spectrophotometer Hitachi (Hitachi, Tokyo, Japan), in a wavelength range from 400 nm to 800 nm. UV spectra of **1**–**18** peptides are presented in Supplementary Materials: Figures S37–S54.

Fluorescence measurements. Performed on FLUOROMAX-3 Horiba Scientific (Edison, NJ, USA) in a wavelength range from 470 nm to 600 nm, excitation wavelength 440 nm. Fluorescence intensity spectra of **1**–**18** peptides are presented in Supplementary Materials: Figures S56–S73.

### 3.3. Spectroscopic Measurements with Congo Red (GP 5) 

To initiate the aggregation process, peptide samples were incubated for 7 days at 37.4 °C in 1 mL of phosphate buffer solution (concentration 0.1 M, pH 7.2), when difficulties in the solubility of peptides in the buffer were observed, samples were sonicated for 15 s. The final concentration of the incubated peptides was c = 1.44 mM. Subsequently, 1 mL of Congo Red (Sigma-Aldrich) solution (c = 45 µM, phosphate buffer, pH 7.2) was added to samples, which were incubated for a further 4 days at room temperature. During this period spectroscopic measurements were performed in the wavelength range of 800 nm to 400 nm. Aggregation studies of all the incubated samples spectroscopic measurements were begun 30 min after the addition of the Congo Red solution. Registered spectra of a mixture containing 1 mL solution of Congo Red (c = 45 µM, phosphate buffer, pH 7.2) and of a 1 mL of solution of phosphate buffer (concentration 0.1 M, pH 7.2), also incubated for 4 days at room temperature, were used as controls. All UV–Vis spectra peptides **1**–**18** incubated with Congo Red are presented in Supplementary Materials. 

### 3.4. Spectroscopic Measurements with Thioflavin T (GP 6) 

To initiate the aggregation process, peptide samples were incubated for 7 days at 37.4 °C in 2 mL of solution of phosphate buffer (concentration 0.1 M, pH 6.0), when difficulties in the solubility of peptides in the buffer were observed, samples were sonicated for 15 s. To the samples was then added 2 mL of Thioflavin T solution (c = 57 mM, phosphate buffer, pH 6.0). The samples were incubated for another 4 days at room temperature. The final concentration of the incubated peptides was c = 0.139 mM. Starting 30 min after the addition of Thioflavin T solution, over the following days fluorescence measurements were performed in the wavelength range from 470 nm to 600 nm (excitation λ = 440 nm). Registered spectra of the mixture containing 2 mL solution of Thioflavin T (c = 57 mM, phosphate buffer, pH 6.0) and 2 mL of phosphate buffer solution (concentration 0.1 M, pH 6.0), also incubated for 4 days at room temperature, were used as the controls. All fluorescence spectra peptides **1**–**18** incubated with Thioflavin T are presented in Supplementary Materials. 

### 3.5. Microscopic Measurements (GP 7)

A sample for microscopic analysis (peptide and Congo Red solution) was centrifuged at 12,000–14,000 rpm in a centrifuge tube to pellet the fibrils, then washed three times with water. The fibrils were then suspended in a small amount of water and placed on a glass microscope slide. The sample was air-dried and analyzed under non-polarized and polarized light using a Delta Optical Genetic Pro microscope (Warsaw, Poland). 

### 3.6. AFM Studies

Atomic force microscopy (AFM) measurements were conducted in tapping mode using an NT-MDT Solver PRO microscope (NT-MDT Spectrum Instruments, Moscow, Russia). Droplets of the peptide samples were placed on glass holders, then dried in air for 5 h. Imaging was performed in air using microfabricated silicon cantilevers (model TESP, force constant about 40 N/m) (NT-MDT Spectrum Instruments, Moscow, Russia). The imaging software was NanoScope Software Version 5.

### 3.7. CD Studies 

CD studies were performed with a Jasco J-1500 spectrometer (ABL and E-JASCO Polska, Cracow, Poland). Far-UV CD experiments were performed using a J-1500 CD spectrometer (Jasco). The samples were prepared in a phosphate buffer solution, pH 7.2, concentration 0.1 mg/mL (1 mg of each analyzed peptide was dissolved in 10 mL of buffer solution). All studies were carried out at ambient temperature. The dissolved samples were loaded in a rectangular quartz cuvette (1 mm path length, Hellma). Spectra were collected in the range of 190–270 nm. Other experimental settings were as follows: Data pitch, 5 nm; scanning mode, continuous; scanning speed, 100 nm/min; bandwidth, 3 nm; integration time, 1 s.

## 4. Conclusions

The research presented here revealed new regions in the structure of insulin that may be involved in its aggregation. Its findings could enable the more rational design of inhibitors in the hormone aggregation process, which is crucial not only from the point of view of developing new drugs for the treatment of diabetes, but also of designing additives for stabilizing insulin preparations. As reference points, two fragments A13–A19 H–LYQLENY–OH (**16**), B12–B17 H–VEALYL–OH (**17**), which are known hot spots of insulin and a fragment B22–B27 H–RGFFYT–OH (**18**) suspected of amyloidogenic properties were used.

It was found that the synthesized peptide **4** (A11–A21) covering peptide **16** was able to aggregate. However, it is surprising that peptides **5** (A11–A16) and **6** (A17–A21) showed different aggregation susceptibility. Fragment A11–A16 can be classified as aggregable (based on a positive ThT test result and microscopic examination with an ambiguous CR assay result). However, fragment A17–A21 (peptide **6**) forms mainly amorphous structures in the aggregation process, instead of the expected fibrous structures.

In the case of the N-terminal fragment of the A-chain, for which di-peptide A3–A4 was pointed as a fragment influencing aggregation, we found that penta-peptides **2** and **3** comprising fragment 1–10 meet all the criteria aggregating peptides. Regarding the B-chain of insulin, for which the B12–B17 fragment is a known hot spot and the peptide susceptible to aggregation is the B22–B27 fragment, varying aggregation ability was also observed in the B11–B20 region (peptides **10**, **11** and **12**). For deca-peptide **10** (B11–B20) and penta-peptide **11** (B11–B15), microscopic studies revealed the formation of fibrous structures. In the case of peptide **12** (B16–B20), microscopic images showed the presence of amorphous and fibrous structures. In microscopic studies of the C-terminal region of the B chain of insulin (peptides **13**, **14** and **15**), sole fiber structures were seen in the case of fragment B21–B25. The CR and ThT assays gave clear results confirming its susceptibility to aggregation. In the N-terminal region of the B chain of insulin for peptides **7** (B1–B10) and **8** (B1–B5), characteristic fibrous structures were observed in microscopic studies. However, all tests (microscopic examination, CR and ThT test) were positive only in the case of peptide **8**. This finding is consistent with the modeling result indicating that fragment B1–B5 may affect insulin aggregation. For deca-peptide **7** (B1–B10) and penta-peptide **9** (B6–B10), the results of three independent tests were ambiguous. CD studies showed that mature fibrils are formed via α-helix rich structures from random coil and immature aggregates. This observation is similar to what has been observed for β-amyloid. Slow and gentle changes were visible in the spectra, from random coils to more β rich structures. These changes were most marked in fragments from chain B: B6–B10, B11–B20 and B1–B5.

## Figures and Tables

**Table 1:** Designed fragments of human insulin covering the entire hormone structure.

Chain A	Chain B
A1–A10 H–GIVEQCCTSI–OH (**1**)	B1–B10 H–FVNQHLCGSH–OH (**7**)
A1–A5 H–GIVEQ–OH (**2**)	B1–B5 H–FVNQH–OH (**8**)
A6–A10 H–CCTSI–OH (**3**)	B6–B10 H–LCGSH–OH (**9**)
A11–A21 H–CSLYQLENYCN–OH (**4**)	B11–B20 H–LVEALYLVCG–OH (**10**)
A11–A16 H–CSLYQL–OH (**5**)	B11–B15 H–LVEAL–OH (**11**)
A17–A21 H–ENYCN–OH (**6**)	B16–B20 H–YLVCG–OH (**12**)
	B21–B30 H–ERGFFYTPKT–OH (**13**)
	B21–B25 H–ERGFF–OH (**14**)
	B26–B30 H–YTPKT–OH (**15**)

**Table 2:** Susceptibility to aggregation of peptides **1**–**15**.

Insulin Fragments	CR Assay	ThT Assay	Microscopic Examination, Morphology
Known Hot Spots of Insulin or Fragment Suspected of Having Amyloidogenic Properties			
A13–A19 H–LYQLENY–OH (**16**)	++	++	++
B12–B17 H–VEALYL–OH (**17**)	++	+	++
B22–B27 H–RGFFYT–OH (**18**)	++	+	+ fibrous structure
**Chain A**			
A1–A10 H–GIVEQCCTSI–OH (**1**)	+	++	+/- cluster of fibrous and amorphous structures
A1–A5 H–GIVEQ–OH (**2**)	+	+	+ fibrous structure
A6–A10 H–CCTSI–OH (**3**)	+	+	+ fibrous structure
A11–A21 H–CSLYQLENYCN–OH (**4**)	+	++	+ fibrous structure
A11–A16 H–CSLYQL–OH (**5**)	+/- almost flat spectrum	+	+ fibrous structure
A17–A21 H–ENYCN–OH (**6**)	+/- almost flat spectrum	+	+/- cluster of fibrous and amorphous structures
**Chain B**			
B1–B10 H–FVNQHLCGSH–OH (**7**)	+	+/-	+ fibrous structure
B1–B5 H–FVNQH–OH (**8**)	+	+	+/- cluster of fibrous and amorphous structures
B6–B10 H–LCGSH–OH (**9**)	+	+/-	+/- cluster of fibrous and amorphous structures
B11–B20 H–LVEALYLVCG–OH (**10**)	+/- almost flat spectrum	+	+ fibrous structure
B11–B15 H–LVEAL–OH (**11**)	+	+	+ fibrous structure
B16–B20 H–YLVCG–OH (**12**)	+/- almost flat spectrum	+	+/- cluster of fibrous and amorphous structures
B21–B30 H–ERGFFYTPKT–OH (**13**)	+/- an absorbance higher than CR, a characteristic shift of the maximum absorbance	+	+/- cluster of fibrous and amorphous structures
B21–B25 H–ERGFF–OH (**14**)	+	+	+ fibrous structure
B26–B30 H–YTPKT–OH (**15**)	+	+/-	+/- cluster of fibrous and amorphous structures

++ very high test result, + positive test result, +/- the result of the test is ambiguous.

## Supplementary Materials

The following are available online at https://www.mdpi.com/1420-3049/24/8/1600/s1, the HPLC of crude **1**–**18** peptides (Figures S1, 3, 5, 7, 9, 11, 13, 15, 17, 19, 21, 23, 25, 27, 29, 31, 33, 35); MS spectra of **1**–**18** peptides (Figures S2, 4, 6, 8, 10, 12, 14, 16, 18, 20, 22, 24, 26, 28, 30, 32, 34, 36); UV spectra of **1**–**18** peptides (Figures S37–S54) and fluorescence intensity spectra of **1**–**18** peptides (Figures S56–S73).
