# Supplementary material for: Search for New Aggregable Fragments of Human Insulin
Source: Molecules. 2019 Apr 23;24(8):1600. doi: 10.3390/molecules24081600 (PMC6514721; doi:10.3390/molecules24081600)
Supplement: Supplementary file 1 [file molecules-24-01600-s001.pdf]

Supporting Material

Search for New Aggregable Fragments of Human Insulin

Monika Swiontek [1], Justyna Fraczyk [1], Joanna Wasko [1], Agata Chaberska [1], Lukasz Pietrzak [2], Zbigniew J. Kaminski [1], Lukasz Szymanski [2], Slawomir Wiak [2] and Beata Kolesinska [1,*]

[1] Institute of Organic Chemistry, Lodz University of Technology, Zeromskiego 116, 90-924 Lodz, Poland; monika.swiontek@gmail.com (M.S.); justyna.fraczyk@p.lodz.pl (J.F.); joanna.wasko@p.lodz.pl (J.W.); agata.chaberska@p.lodz.pl (A.C.); zbigniew.kaminski@p.lodz.pl (Z.J.K.)

[2] Institute of Mechatronics and Information Systems, Faculty of Electrical, Electronic, Computer and Control Engineering, Lodz University of Technology, Stefanowskiego 18/22, 90-924 Lodz, Poland; lukasz.pietrzak@p.lodz.pl (L.P.); lukasz.szymanski@p.lodz.pl (L.S.); slawomir.wiak@p.lodz.pl (S.W.)

* Correspondence: beata.kolesinska@p.lodz.pl; Tel.: +48-42-631-3149

Synthesis of A1-A10 fragment: H-GlyIleValGluGlnCysCysThrSerIle-OH (**1**).

2-Chlorotrityl Chloride resin (0.25 g, 0.25 mol) was estrificated with Fmoc-Ile-OH (88.35 mg; 0.25 mmol) according to *GP1,* followed by Fmoc deprotection (*GP 3*). Subsequently, the peptide chain was elongated with:

Fmoc-Ser(tBu)-OH (253.07 mg; 0.66 mmol) in the presence of DMT/NMM/TosO$^-$ (272.58 mg; 0.66 mmol) and NMM (145.20 μL; 1.32 mmol) (*GP 2*).

Fmoc-Thr(tBu)-OH (262.32 mg; 0.66 mmol) in the presence of DMT/NMM/TosO$^-$ (272.58 mg; 0.66 mmol) and NMM (145.20 μL; 1.32 mmol) (*GP 2*).

Fmoc-Cys(Trt)-OH (386.57 mg; 0.66 mmol) in the presence of DMT/NMM/TosO$^-$ (272.58 mg; 0.66 mmol) and NMM (145.20 μL; 1.32 mmol) (*GP 2*).

Fmoc-Cys(Trt)-OH (386.57 mg; 0.66 mmol) in the presence of DMT/NMM/TosO$^-$ (272.58 mg; 0.66 mmol) and NMM (145.20 μL; 1.32 mmol) (*GP 2*).

Fmoc-Gln(Trt)-OH (403.06 mg; 0.66 mmol) in the presence of DMT/NMM/TosO$^-$ (272.58 mg; 0.66 mmol) and NMM (145.20 μL; 1.32 mmol) (*GP 2*).

Fmoc-Glu(OtBu)-OH (280.81 mg; 0.66 mmol) in the presence of DMT/NMM/TosO$^-$ (272.58 mg; 0.66 mmol) and NMM (145.20 μL; 1.32 mmol) (*GP 2*).

Fmoc-Val-OH (223.99 mg; 0.66 mmol) in the presence of DMT/NMM/TosO$^-$ (272.58 mg; 0.66 mmol) and NMM (145.20 μL; 1.32 mmol) (*GP 2).*

Fmoc-Ile-OH (233.25 mg; 0.66 mmol) in the presence of DMT/NMM/TosO$^-$ (272.58 mg; 0.66 mmol) and NMM (145.20 μL; 1.32 mmol) (*GP 2*).

Fmoc-Gly-OH (196.22 mg; 0.66 mmol) in the presence of DMT/NMM/TosO$^-$ (272.58 mg; 0.66 mmol) and NMM (145.20 μL; 1.32 mmol) (*GP 2*).

After deprotection (*GP 3*), the peptide was cleaved form the resin (*GP 4*). Anal. RP-HPLC (0-5 % A in 30 min.): t_R 16.0 min., purity > 97 % (Fig. 1). LC-MS: 1053.4 $[M+H]^+$, calcd. 1052.24 g/mol (Fig. 2).

Synthesis of A1-A5 fragment:H-GlyIleValGluGln-OH (**2**).

2-Chlorotrityl Chloride resin (0.25 g, 0.25 mol) was estrificated with Fmoc-Gln(Trt)-OH (152.67 mg;0.25 mmol) according to *GP1,* followed by Fmoc deprotection (*GP 3*). Subsequently, the peptide chain was elongated with:

Fmoc-Glu(OtBu)-OH (280.81 mg; 0.66 mmol) in the presence of DMT/NMM/TosO$^-$ (272.58 mg; 0.66 mmol) and NMM (145.20 μL; 1.32 mmol) (*GP 2*).

Fmoc-Val-OH (223.99 mg; 0.66 mmol) in the presence of DMT/NMM/TosO$^-$ (272.58 mg; 0.66 mmol) and NMM (145.20 μL; 1.32 mmol) (*GP 2).*

Fmoc-Ile-OH (233.25 mg; 0.66 mmol) in the presence of DMT/NMM/TosO$^-$ (272.58 mg; 0.66 mmol) and NMM (145.20 μL; 1.32 mmol) (*GP 2*).

Fmoc-Gly-OH (196.22 mg; 0.66 mmol) in the presence of DMT/NMM/TosO$^-$ (272.58 mg; 0.66 mmol) and NMM (145.20 μL; 1.32 mmol) (*GP 2*).

After deprotection (*GP 3*), the peptide was cleaved form the resin (*GP 4*). Anal. RP-HPLC (0-5 % A in 30 min.): t_R 3.0 min., purity > 97 % (Fig. 3). LC-MS: 545.2956 $[M+H]^+$, calcd. 544.598 g/mol (Fig. 4).

Synthesis of A6-A10 fragment: H-CysCysThrSerIle-OH (**3**).

2-Chlorotrityl Chloride resin (0.25 g, 0.25 mol) was estrificated with Fmoc-Ile-OH (88.35 mg; 0.25 mmol) according to *GP1,* followed by Fmoc deprotection (*GP 3*). Subsequently, the peptide chain was elongated with:

Fmoc-Ser(tBu)-OH (253.07 mg; 0.66 mmol) in the presence of DMT/NMM/TosO$^-$ (272.58 mg; 0.66 mmol) and NMM (145.20 μL; 1.32 mmol) (*GP 2*).

Fmoc-Thr(tBu)-OH (262.32 mg; 0.66 mmol) in the presence of DMT/NMM/TosO$^-$ (272.58 mg; 0.66 mmol) and NMM (145.20 μL; 1.32 mmol) (*GP 2*).

Fmoc-Cys(Trt)-OH (386.57 mg; 0.66 mmol) in the presence of DMT/NMM/TosO$^-$ (272.58 mg; 0.66 mmol) and NMM (145.20 μL; 1.32 mmol) (*GP 2*).

Fmoc-Cys(Trt)-OH (386.57 mg; 0.66 mmol) in the presence of DMT/NMM/TosO$^-$ (272.58 mg; 0.66 mmol) and NMM (145.20 μL; 1.32 mmol) (*GP 2*).

After deprotection (*GP 3*), the peptide was cleaved form the resin (*GP 4*). Anal. RP-HPLC (0-5 % A in 30 min.): t_R 17.0 min., purity > 89% (Fig. 5). LC-MS: 525.6531 $[M+H]^+$, calcd. 525.65 g/mol (Fig. 6).

Synthesis of A11-A21 fragment: H-CysSerLeuTyrGlnLeuGluAsnTyrCysAsn-OH (**4**).

2-Chlorotrityl Chloride resin (0.25 g, 0.25 mol) was estrificated with Fmoc-Asn(Trt)-OH (149.17 mg; 0.25 mmol) according to *GP1,* followed by Fmoc deprotection (*GP 3*). Subsequently, the peptide chain was elongated with:

Fmoc-Cys(Trt)-OH (386.57 mg; 0.66 mmol) in the presence of DMT/NMM/TosO$^-$ (272.58 mg; 0.66 mmol) and NMM (145.20 μL; 1.32 mmol) (*GP 2*).

Fmoc-Tyr(tBu)-OH (303.29 mg; 0.66 mmol) in the presence of DMT/NMM/TosO$^-$ (272.58 mg; 0.66 mmol) and NMM (145.20 μL; 1.32 mmol) (*GP 2*).

Fmoc-Asn(Trt)-OH (393.80 mg; 0.66 mmol) in the presence of DMT/NMM/TosO$^-$ (272.58 mg; 0.66 mmol) and NMM (145.20 μL; 1.32 mmol) (*GP 2*).

Fmoc-Glu(OtBu)-OH (280.81 mg; 0.66 mmol) in the presence of DMT/NMM/TosO$^-$ (272.58 mg; 0.66 mmol) and NMM (145.20 μL; 1.32 mmol) (*GP 2*).

Fmoc-Leu-OH (233.25 mg; 0.66 mmol) in the presence of DMT/NMM/TosO$^-$ (272.58 mg; 0.66 mmol) and NMM (145.20 μL; 1.32 mmol) (*GP 2*).

Fmoc-Gln(Trt)-OH (403.06 mg; 0.66 mmol) in the presence of DMT/NMM/TosO$^-$ (272.58 mg; 0.66 mmol) and NMM (145.20 μL; 1.32 mmol) (*GP 2*).

Fmoc-Tyr(tBu)-OH (303.29 mg; 0.66 mmol) in the presence of DMT/NMM/TosO$^-$ (272.58 mg; 0.66 mmol) and NMM (145.20 μL; 1.32 mmol) (*GP 2*).

Fmoc-Leu-OH (233.25 mg; 0.66 mmol) in the presence of DMT/NMM/TosO$^-$ (272.58 mg; 0.66 mmol) and NMM (145.20 μL; 1.32 mmol) (*GP 2*).

Fmoc-Ser(tBu)-OH (253.07 mg; 0.66 mmol) in the presence of DMT/NMM/TsO$^-$ (272.58 mg; 0.66 mmol) and NMM (145.20 μL; 1.32 mmol) (*GP 2*).

Fmoc-Cys(Trt)-OH (386.57 mg; 0.66 mmol) in the presence of DMT/NMM/TosO$^-$ (272.58 mg; 0.66 mmol) and NMM (145.20 μL; 1.32 mmol) (*GP 2*).

After deprotection (*GP 3*), the peptide was cleaved form the resin (*GP 4*). Anal. RP-HPLC (0-5 % A in 30 min.): t_R 17.5 min., purity > 85% (Fig. 7). LC-MS: 1348.4 [M+H]$^+$, calcd. 1349.51 g/mol (Fig. 8).

Synthesis of A11-A16 fragment: H-CysSerLeuTyrGlnLeu-OH (**5**).

2-Chlorotrityl Chloride resin (0.25 g, 0.25 mol) was estrificated with Fmoc-Leu-OH (88.35 mg; 0.25 mmol) according to *GP1,* followed by Fmoc deprotection (*GP 3*). Subsequently, the peptide chain was elongated with:

Fmoc-Gln(Trt)-OH (403.06 mg; 0.66 mmol) in the presence of DMT/NMM/TosO$^-$ (272.58 mg; 0.66 mmol) and NMM (145.20 μL; 1.32 mmol) (*GP 2*).

Fmoc-Tyr(tBu)-OH (303.29 mg; 0.66 mmol) in the presence of DMT/NMM/TosO$^-$ (272.58 mg; 0.66 mmol) and NMM (145.20 μL; 1.32 mmol) (*GP 2*).

Fmoc-Leu-OH (233.25 mg; 0.66 mmol) in the presence of DMT/NMM/TosO$^-$ (272.58 mg; 0.66 mmol) and NMM (145.20 μL; 1.32 mmol) (*GP 2*).

Fmoc-Ser(tBu)-OH (253.07 mg; 0.66 mmol) in the presence of DMT/NMM/TosO$^-$ (272.58 mg; 0.66 mmol) and NMM (145.20 μL; 1.32 mmol) (*GP 2*).

Fmoc-Cys(Trt)-OH (386.57 mg; 0.66 mmol) in the presence of DMT/NMM/TosO$^-$ (272.58 mg; 0.66 mmol) and NMM (145.20 μL; 1.32 mmol) (*GP 2*).

After deprotection (*GP 3*), the peptide was cleaved form the resin (*GP 4*). Anal. RP-HPLC (0-5 % A in 30 min.): t_R 15.5 min., purity >95% (Fig. 9). LC-MS: 725.9762 [M+H]$^+$, calcd. 725.87 g/mol (Fig. 10).

Synthesis of A17-A21 fragment: H-GluAsnTyrCysAsn-OH (**6**).

2-Chlorotrityl Chloride resin (0.25 g, 0.25 mol) was estrificated with Fmoc-Asn(Trt)-OH (149.17 mg; 0.25 mmol) according to *GP1,* followed by Fmoc deprotection (*GP 3*). Subsequently, the peptide chain was elongated with:

Fmoc-Cys(Trt)-OH (386.57 mg; 0.66 mmol) in the presence of DMT/NMM/TosO$^-$ (272.58 mg; 0.66 mmol) and NMM (145.20 μL; 1.32 mmol) (*GP 2*).

Fmoc-Tyr(tBu)-OH (303.29 mg; 0.66 mmol) in the presence of DMT/NMM/TosO$^-$ (272.58 mg; 0.66 mmol) and NMM (145.20 μL; 1.32 mmol) (*GP 2*).

Fmoc-Asn(Trt)-OH (393.80 mg; 0.66 mmol) in the presence of DMT/NMM/TosO$^-$ (272.58 mg; 0.66 mmol) and NMM (145.20 μL; 1.32 mmol) (*GP 2*).

Fmoc-Glu(OtBu)-OH (280.81 mg; 0.66 mmol) in the presence of DMT/NMM/TosO$^-$ (272.58 mg; 0.66 mmol) and NMM (145.20 μL; 1.32 mmol) (*GP 2*).

After deprotection (*GP 3*), the peptide was cleaved form the resin (*GP 4*). Anal. RP-HPLC (0-5 % A in 30 min.): t_R 20.5 min., purity >90 % (Fig. 11). LC-MS: 640.6 [M+H]$^+$, calcd. 641.66 g/mol (Fig. 12).

Synthesis of B1-B10 fragment: H-PheValAsnGlnHisLeuCysGlySerHis-OH (**7**).

2-Chlorotrityl Chloride resin (0.25 g, 0.25 mol) was estrificated with Fmoc-His(Trt)-OH (154.93 mg; 0.25 mmol) according to *GP1,* followed by Fmoc deprotection (*GP 3*). Subsequently, the peptide chain was elongated with:

Fmoc-Ser(tBu)-OH (253.07 mg; 0.66 mmol) in the presence of DMT/NMM/TosO$^-$ (272.58 mg; 0.66 mmol) and NMM (145.20 μL; 1.32 mmol) (*GP 2*).

Fmoc-Gly-OH (196.22 mg; 0.66 mmol) in the presence of DMT/NMM/TosO$^-$ (272.58 mg; 0.66 mmol) and NMM (145.20 μL; 1.32 mmol) (*GP 2*).

Fmoc-Cys(Trt)-OH (386.57 mg; 0.66 mmol) in the presence of DMT/NMM/TosO$^-$ (272.58 mg; 0.66 mmol) and NMM (145.20 μL; 1.32 mmol) (*GP 2*).

Fmoc-Leu-OH (233.25 mg; 0.66 mmol) in the presence of DMT/NMM/TosO$^-$ (272.58 mg; 0.66 mmol) and NMM (145.20 μL; 1.32 mmol) (*GP 2*).

Fmoc-His(Trt)-OH (409.01 mg; 0.66 mmol) in the presence of DMT/NMM/TosO$^-$ (272.58 mg; 0.66 mmol) and NMM (145.20 μL; 1.32 mmol) (*GP 2*).

Fmoc-Gln(Trt)-OH (403.06 mg; 0.66 mmol) in the presence of DMT/NMM/TosO$^-$ (272.58 mg; 0.66 mmol) and NMM (145.20 μL; 1.32 mmol) (*GP 2*).

Fmoc-Asn(Trt)-OH (393.80 mg; 0.66 mmol) in the presence of DMT/NMM/TosO$^-$ (272.58 mg; 0.66 mmol) and NMM (145.20 μL; 1.32 mmol) (*GP 2*).

Fmoc-Val-OH (223.10 mg; 0.66 mmol) in the presence of DMT/NMM/TosO$^-$ (272.58 mg; 0.66 mmol) and NMM (145.20 μL; 1.32 mmol) (*GP 2*).

Fmoc-Phe-OH (255.70 mg; 0.66 mmol) in the presence of DMT/NMM/TosO$^-$ (272.58 mg; 0.66 mmol) and NMM (145.20 μL; 1.32 mmol) (*GP 2*).

After deprotection (*GP 3*), the peptide was cleaved form the resin (*GP 4*). Anal. RP-HPLC (0-5 % A in 30 min.): t_R 21.5 min., purity >98 % (Fig. 13). LC-MS: 1142.6 [M+H]$^+$, calcd. 1141.28 g/mol (Fig. 14).

Synthesis of B1-B5 fragment: H-PheValAsnGlnHis-OH (**8**).

2-Chlorotrityl Chloride resin (0.25 g, 0.25 mol) was estrificated with Fmoc-His(Trt)-OH (154.93 mg; 0.25 mmol) according to *GP1,* followed by Fmoc deprotection (*GP 3*). Subsequently, the peptide chain was elongated with:

Fmoc-Gln(Trt)-OH (403.06 mg; 0.66 mmol) in the presence of DMT/NMM/TosO$^-$ (272.58 mg; 0.66 mmol) and NMM (145.20 μL; 1.32 mmol) (*GP 2*).

Fmoc-Asn(Trt)-OH (393.80 mg; 0.66 mmol) in the presence of DMT/NMM/TosO$^-$ (272.58 mg; 0.66 mmol) and NMM (145.20 μL; 1.32 mmol) (*GP 2*).

Fmoc-Val-OH (223.10 mg; 0.66 mmol) in the presence of DMT/NMM/TosO$^-$ (272.58 mg; 0.66 mmol) and NMM (145.20 μL; 1.32 mmol) (*GP 2*).

Fmoc-Phe-OH (255.70 mg; 0.66 mmol) in the presence of DMT/NMM/TosO$^-$ (272.58 mg; 0.66 mmol) and NMM (145.20 μL; 1.32 mmol) (*GP 2*).

After deprotection (*GP 3*), the peptide was cleaved form the resin (*GP 4*). Anal. RP-HPLC (0-5 % A in 30 min.): t_R 2.9 min., purity >95 % (Fig. 15). LC-MS: 644.3168 [M+H]$^+$, calcd. 643.69 g/mol (Fig. 16).

Synthesis of B6-B10 fragment of human insulin H-Leu-Cys-Gly-Ser-His-OH (**9**).

2-Chlorotrityl Chloride resin (0.25 g, 0.25 mol) was estrificated with Fmoc-His(Trt)-OH (154.93 mg; 0.25 mmol) according to *GP1,* followed by Fmoc deprotection (*GP 3*). Subsequently, the peptide chain was elongated with:

Fmoc-Ser(tBu)-OH (253.04 mg; 0.66 mmol) in the presence of DMT/NMM/TosO$^-$ (272.58 mg; 0.66 mmol) and NMM (145.20 μL; 1.32 mmol) (*GP 2*).

Fmoc-Gly-OH (196.22 mg; 0.66 mmol) in the presence of DMT/NMM/TosO$^-$ (272.58 mg; 0.66 mmol) and NMM (145.20 μL; 1.32 mmol) (*GP 2*).

Fmoc-Cys(Trt)-OH (386.57 mg; 0.66 mmol) in the presence of DMT/NMM/TosO$^-$ (272.58 mg; 0.66 mmol) and NMM (145.20 μL; 1.32 mmol) (*GP 2*).

Fmoc-Leu-OH (233.26 mg; 0.66 mmol) in the presence of DMT/NMM/TosO$^-$ (272.58 mg; 0.66 mmol) and NMM (145.20 µL; 1.32 mmol) (*GP 2*).

After deprotection (*GP 3*), the peptide was cleaved form the resin (*GP 4*). Anal. RP-HPLC (0-5 % A in 30 min.): t_R 15 min., purity > 85% (Fig. 17). LC-MS: 513.8 [M+H]$^+$, calcd. 515.59 g/mol (Fig. 18).

Synthesis of B11-B20 fragment: H-LeuValGluAlaLeuTyrLeuValCysGly-OH (**10**).

2-Chlorotrityl Chloride resin (0.25 g, 0.25 mol) was estrificated with Fmoc-Gly-OH (74.33 mg; 0.25 mmol) according to *GP1,* followed by Fmoc deprotection (*GP 3*). Subsequently, the peptide chain was elongated with:

Fmoc-Cys(Trt)-OH (386.57 mg; 0.66 mmol) in the presence of DMT/NMM/TosO$^-$ (272.58 mg; 0.66 mmol) and NMM (145.20 µL; 1.32 mmol) (*GP 2*).

Fmoc-Val-OH (223.10 mg; 0.66 mmol) in the presence of DMT/NMM/TosO$^-$ (272.58 mg; 0.66 mmol) and NMM (145.20 µL; 1.32 mmol) (*GP 2*).

Fmoc-Leu-OH (233.25 mg; 0.66 mmol) in the presence of DMT/NMM/TosO$^-$ (272.58 mg; 0.66 mmol) and NMM (145.20 µL; 1.32 mmol) (*GP 2*).

Fmoc-Tyr(tBu)-OH (303.29 mg; 0.66 mmol) in the presence of DMT/NMM/TosO$^-$ (272.58 mg; 0.66 mmol) and NMM (145.20 µL; 1.32 mmol) (*GP 2*).

Fmoc-Leu-OH (233.25 mg; 0.66 mmol) in the presence of DMT/NMM/TosO$^-$ (272.58 mg; 0.66 mmol) and NMM (145.20 µL; 1.32 mmol) (*GP 2*).

Fmoc-Ala-OH (205.48 mg; 0.66 mmol) in the presence of DMT/NMM/TosO$^-$ (272.58 mg; 0.66 mmol) and NMM (145.20 µL; 1.32 mmol) (*GP 2*).

Fmoc-Glu(OtBu)-OH (280.81 mg; 0.66 mmol) in the presence of DMT/NMM/TosO$^-$ (272.58 mg; 0.66 mmol) and NMM (145.20 µL; 1.32 mmol) (*GP 2*).

Fmoc-Val-OH (223.10 mg; 0.66 mmol) in the presence of DMT/NMM/TosO$^-$ (272.58 mg; 0.66 mmol) and NMM (145.20 µL; 1.32 mmol) (*GP 2*).

Fmoc-Leu-OH (233.25 mg; 0.66 mmol) in the presence of DMT/NMM/TosO$^-$ (272.58 mg; 0.66 mmol) and NMM (145.20 µL; 1.32 mmol) (*GP 2*).

After deprotection (*GP 3*), the peptide was cleaved form the resin (*GP 4*). Anal. RP-HPLC (0-5 % A in 30 min.): t_R 19.1 min., purity > 89% (Fig. 19). LC-MS: 1079.6 [M+H]$^+$, calcd. 1079.33 g/mol (Fig. 20).

Synthesis of B11-B15 fragment: H-LeuValGluAlaLeu-OH (**11**).

2-Chlorotrityl Chloride resin (0.25 g, 0.25 mol) was estrificated with Fmoc-Leu-OH (88.35 mg; 0.25 mmol) according to *GP1,* followed by Fmoc deprotection (*GP 3*). Subsequently, the peptide chain was elongated with:

Fmoc-Ala-OH (205.48 mg; 0.66 mmol) in the presence of DMT/NMM/TosO$^-$ (272.58 mg; 0.66 mmol) and NMM (145.20 μL; 1.32 mmol) (*GP 2*).

Fmoc-Glu(OtBu)-OH (280.81 mg; 0.66 mmol) in the presence of DMT/NMM/TosO$^-$ (272.58 mg; 0.66 mmol) and NMM (145.20 μL; 1.32 mmol) (*GP 2*).

Fmoc-Val-OH (223.10 mg; 0.66 mmol) in the presence of DMT/NMM/TosO$^-$ (272.58 mg; 0.66 mmol) and NMM (145.20 μL; 1.32 mmol) (*GP 2*).

Fmoc-Leu-OH (233.26 mg; 0.66 mmol) in the presence of DMT/NMM/TosO$^-$ (272.58 mg; 0.66 mmol) and NMM (145.20 μL; 1.32 mmol) (*GP 2*).

After deprotection (*GP 3*), the peptide was cleaved form the resin (*GP 4*). Anal. RP-HPLC (0-5 % A in 30 min.): t_R 16.3 min., purity >98 % (Fig. 21). LC-MS: 544.4254 [M+H]$^+$, calcd. 543.67 g/mol (Fig. 22).

Synthesis of B16-B20 fragment: H-TyrLeuValCysGly-OH (**12**).

2-Chlorotrityl Chloride resin (0.25 g, 0.25 mol) was estrificated with Fmoc-Gly-OH (74.33 mg; 0.25 mmol) according to *GP1,* followed by Fmoc deprotection (*GP 3*). Subsequently, the peptide chain was elongated with:

Fmoc-Cys(Trt)-OH (386.57 mg; 0.66 mmol) in the presence of DMT/NMM/TosO$^-$ (272.58 mg; 0.66 mmol) and NMM (145.20 μL; 1.32 mmol) (*GP o*).

Fmoc-Val-OH (223.10 mg; 0.66 mmol) in the presence of DMT/NMM/TosO$^-$ (272.58 mg; 0.66 mmol) and NMM (145.20 μL; 1.32 mmol) (*GP o*).

Fmoc-Leu-OH (223.26 mg; 0.66 mmol) in the presence of DMT/NMM/TosO$^-$ (272.58 mg; 0.66 mmol) and NMM (145.20 μL; 1.32 mmol) (*GP 2*).

Fmoc-Tyr(tBu)-OH (303.29 mg; 0.66 mmol) in the presence of DMT/NMM/TosO$^-$ (272.58 mg; 0.66 mmol) and NMM (145.20 μL; 1.32 mmol) (*GP 2*).

After deprotection (*GP 3*), the peptide was cleaved form the resin (*GP 4*). Anal. RP-HPLC (0-5 % A in 30 min.): t_R 14.2 min., purity >71 % (Fig. 23). LC-MS: 553.9 [M+H]$^+$, calcd. 553.68 g/mol (Fig. 24).

Synthesis of B21-B30 fragment: H-GluArgGlyPhePheTyrThrProLysThr-COOH (**13**).

2-Chlorotrityl Chloride resin (0.25 g, 0.25 mol) was estrificated with Fmoc-Thr(tBu)-OH (99.37 mg; 0.25 mmol) according to *GP1,* followed by Fmoc deprotection (*GP 3*). Subsequently, the peptide chain was elongated with:

Fmoc-Lys(Boc)-OH (309.24 mg; 0.66 mmol) in the presence of DMT/NMM/TosO$^-$ (272.58 mg; 0.66 mmol) and NMM (145.20 μL; 1.32 mmol) (*GP 2*).

Fmoc-Pro-OH (222.66 mg; 0.66 mmol) in the presence of DMT/NMM/TosO$^-$ (272.58 mg; 0.66 mmol) and NMM (145.20 μL; 1.32 mmol) (*GP 2*).

Fmoc-Thr(tBu)-OH (262.32 mg; 0.66 mmol) in the presence of DMT/NMM/TosO$^-$ (272.58 mg; 0.66 mmol) and NMM (145.20 µL; 1.32 mmol) (*GP 2*).

Fmoc-Tyr(tBu)-OH (303.29 mg; 0.66 mmol) in the presence of DMT/NMM/TosO$^-$ (272.58 mg; 0.66 mmol) and NMM (145.20 µL; 1.32 mmol) (*GP 2*).

Fmoc-Phe-OH (255.70 mg; 0.66 mmol) in the presence of DMT/NMM/TosO$^-$ (272.58 mg; 0.66 mmol) and NMM (145.20 µL; 1.32 mmol) (*GP 2*).

Fmoc-Phe-OH (255.70 mg; 0.66 mmol) in the presence of DMT/NMM/TosO$^-$ (272.58 mg; 0.66 mmol) and NMM (145.20 µL; 1.32 mmol) (*GP 2*).

Fmoc-Gly-OH (196.22 mg; 0.66 mmol) in the presence of DMT/NMM/TosO$^-$ (272.58 mg; 0.66 mmol) and NMM (145.20 µL; 1.32 mmol) (*GP 2*).

Fmoc-Arg(Pbf)-OH (428.19 mg; 0.66 mmol) in the presence of DMT/NMM/TosO$^-$ (272.58 mg; 0.66 mmol) and NMM (145.20 µL; 1.32 mmol) (*GP 2*).

Fmoc-Glu(OtBu)-OH (280.81mg; 0.66 mmol) in the presence of DMT/NMM/TosO$^-$ (272.58 mg; 0.66 mmol) and NMM (145.20 µL; 1.32 mmol) (*GP 2*).

After deprotection (*GP 2*), the peptide was cleaved form the resin (*GP 4*). Anal. RP-HPLC (0-5 % A in 30 min.): t_R 10 min., purity >94 % (Fig. 25). LC-MS: 1245.6119 [M+H]$^+$, calcd. 1245.41 g/mol (Fig. 26).

Synthesis of B21-B25 fragment: H-GluArgGlyPhePhe-COOH (**14**).

2-Chlorotrityl Chloride resin (0.25 g, 0.25 mol) was estrificated with Fmoc-Phe-OH (96.86 mg; 0.25 mmol) according to *GP1,* followed by Fmoc deprotection (*GP 3*). Subsequently, the peptide chain was elongated with:

Fmoc-Phe-OH (255.77 mg; 0.66 mmol) in the presence of DMT/NMM/TosO$^-$ (272.58 mg; 0.66 mmol) and NMM (145.20 µL; 1.32 mmol) (*GP 2*).

Fmoc-Gly-OH (196.22 mg; 0.66 mmol) in the presence of DMT/NMM/TosO$^-$ (272.58 mg; 0.66 mmol) and NMM (145.20 µL; 1.32 mmol) (*GP 2*).

Fmoc-Arg(Pbf)-OH (428.19 mg; 0.66 mmol) in the presence of DMT/NMM/TosO$^-$ (272.58 mg; 0.66 mmol) and NMM (145.20 µL; 1.32 mmol) (*GP 2*).

Fmoc-Glu(OtBu)-OH (280.8 mg; 0.66 mmol) in the presence of DMT/NMM/TosO$^-$ (272.58 mg; 0.66 mmol) and NMM (145.20 µL; 1.32 mmol) (*GP 2*).

After deprotection (*GP 3*), the peptide was cleaved form the resin (*GP 4*). Anal. RP-HPLC (0-5 % A in 30 min.): t_R 15 min., purity >71 % (Fig. 27). LC-MS: 655.3216 [M+H]$^+$, calcd. 654.73 g/mol (Fig. 28).

Synthesis of B26-B30 fragment: H-TyrThrProLysThr-OH (**15**).

2-Chlorotrityl Chloride resin (0.25 g, 0.25 mol) was estrificated with Fmoc-Thr(tBu)-OH (114.88 mg; 0.25 mmol) according to *GP1,* followed by Fmoc deprotection (*GP 3*). Subsequently, the peptide chain was elongated with:

Fmoc-Lys(Boc)-OH (309.24 mg; 0.66 mmol) in the presence of DMT/NMM/TosO$^-$ (272.58 mg; 0.66 mmol) and NMM (145.20 μL; 1.32 mmol) (*GP 2*).

Fmoc-Pro-OH (222.66 mg; 0.66 mmol) in the presence of DMT/NMM/TosO$^-$ (272.58 mg; 0.66 mmol) and NMM (145.20 μL; 1.32 mmol) (*GP 2*).

Fmoc-Thr(tBu)-OH (262.32 mg; 0.66 mmol) in the presence of DMT/NMM/TosO$^-$ (272.58 mg; 0.66 mmol) and NMM (145.20 μL; 1.32 mmol) (*GP 2*).

Fmoc-Tyr(tBu)-OH (303.29 mg; 0.66 mmol) in the presence of DMT/NMM/TosO$^-$ (272.58 mg; 0.66 mmol) and NMM (145.20 μL; 1.32 mmol) (*GP 2*).

After deprotection (*GP 3*, the peptide was cleaved form the resin (*GP 4*). Anal. RP-HPLC (0-5 % A in 30 min.): t_R 23.813 min., purity >83 % (Fig. 29). LC-MS: 608.4574 $[M+H]^+$, calcd. 608.70 g/mol (Fig. 30).

Synthesis of A13-A19 fragment: H-LeuTyrGlnLeuGluAsnTyr-OH (**16**).

2-Chlorotrityl Chloride resin (0.25 g, 0.25 mol) was estrificated with Fmoc-Tyr(tBu)-OH (114.88 mg; 0.25 mmol) according to *GP1,* followed by Fmoc deprotection (*GP 3*). Subsequently, the peptide chain was elongated with:

Fmoc-Asn(Trt)-OH (393.80 mg; 0.66 mmol) in the presence of DMT/NMM/TosO$^-$ (272.58 mg; 0.66 mmol) and NMM (145.20 μL; 1.32 mmol) (*GP 2*).

Fmoc-Glu(OtBu)-OH (280.81 mg; 0.66 mmol) in the presence of DMT/NMM/TosO$^-$ (272.58 mg; 0.66 mmol) and NMM (145.20 μL; 1.32 mmol) (*GP 2*).

Fmoc-Leu-OH (233.25 mg; 0.66 mmol) in the presence of DMT/NMM/TosO$^-$ (272.58 mg; 0.66 mmol) and NMM (145.20 μL; 1.32 mmol) (*GP 2*).

Fmoc-Gln(Trt)-OH (403.06 mg; 0.66 mmol) in the presence of DMT/NMM/TosO$^-$ (272.58 mg; 0.66 mmol) and NMM (145.20 μL; 1.32 mmol) (*GP 2*).

Fmoc-Leu-OH (233.25 mg; 0.66 mmol) in the presence of DMT/NMM/TosO$^-$ (272.58 mg; 0.66 mmol) and NMM (145.20 μL; 1.32 mmol) (*GP 2*).

After deprotection (*GP 3*), the peptide was cleaved form the resin (*GP 4*). Anal. RP-HPLC (0-5 % A in 30 min.): t_R 3.948 min., purity >94 % (Fig. 31). LC-MS: 942.8 $[M+H]^+$, calcd. 942.8 g/mol (Fig. 32).

Synthesis of B12-B17 fragment: H-ValGluAlaLeuTyrLeu-OH (**17**).

2-Chlorotrityl Chloride resin (0.25 g, 0.25 mol) was estrificated with Fmoc-Leu-OH (88.35 mg; 0.25 mmol) according to *GP1,* followed by Fmoc deprotection (*GP 3*). Subsequently, the peptide chain was elongated with:

Fmoc-Tyr(tBu)-OH (303.29 mg; 0.66 mmol) in the presence of DMT/NMM/TosO$^-$ (272.58 mg; 0.66 mmol) and NMM (145.20 µL; 1.32 mmol) (*GP 2*).

Fmoc-Leu-OH (233.25 mg; 0.66 mmol) in the presence of DMT/NMM/TosO$^-$ (272.58 mg; 0.66 mmol) and NMM (145.20 µL; 1.32 mmol) (*GP 2*).

Fmoc-Ala-OH (205.48 mg; 0.66 mmol) in the presence of DMT/NMM/TosO$^-$ (272.58 mg; 0.66 mmol) and NMM (145.20 µL; 1.32 mmol) (*GP 2*).

Fmoc-Glu(OtBu)-OH (280.81 mg; 0.66 mmol) in the presence of DMT/NMM/TosO$^-$ (272.58 mg; 0.66 mmol) and NMM (145.20 µL; 1.32 mmol) (*GP 2*).

Fmoc-Val-OH (223.10 mg; 0.66 mmol) in the presence of DMT/NMM/TosO$^-$ (272.58 mg; 0.66 mmol) and NMM (145.20 µL; 1.32 mmol) (*GP 2*).

After deprotection (*GP 3*), the peptide was cleaved form the resin (*GP 4*). Anal. RP-HPLC (0-5 % A in 30 min.): t_R 21.74 min., purity >99.31 % (Fig. 33). LC-MS: 707.5 $[M+H]^+$, calcd. 706.84 g/mol (Fig. 34).

Synthesis of B22-B27 fragment: H-ArgGlyPhePheTyrThr-OH (**18**).

2-Chlorotrityl Chloride resin (0.25 g, 0.25 mol) was estrificated with Fmoc-Thr(tBu)-OH (99.37 mg; 0.25 mmol) according to *GP1,* followed by Fmoc deprotection (*GP 3*). Subsequently, the peptide chain was elongated with:

Fmoc-Tyr(tBu)-OH (303.29 mg; 0.66 mmol) in the presence of DMT/NMM/TosO$^-$ (272.58 mg; 0.66 mmol) and NMM (145.20 µL; 1.32 mmol) (*GP 2*).

Fmoc-Phe-OH (255.70 mg; 0.66 mmol) in the presence of DMT/NMM/TosO$^-$ (272.58 mg; 0.66 mmol) and NMM (145.20 µL; 1.32 mmol) (*GP 2*).

Fmoc-Phe-OH (255.70 mg; 0.66 mmol) in the presence of DMT/NMM/TosO$^-$ (272.58 mg; 0.66 mmol) and NMM (145.20 µL; 1.32 mmol) (*GP 2*).

Fmoc-Gly-OH (196.23 mg; 0.66 mmol) in the presence of DMT/NMM/TosO$^-$ (272.58 mg; 0.66 mmol) and NMM (145.20 µL; 1.32 mmol) (*GP 2*).

Fmoc-Arg(Pbf)-OH (428.19 mg; 0.66 mmol) in the presence of DMT/NMM/TosO$^-$ (272.58 mg; 0.66 mmol) and NMM (145.20 µL; 1.32 mmol) (*GP 2*).

After deprotection (*GP 3*), the peptide was cleaved form the resin (*GP 3*). Anal. RP-HPLC (0-5 % A in 30 min.): t_R 7.595 min., purity >85 % (Fig. 35). LC-MS: 789.5047 $[M+H]^+$, calcd. 788.89 g/mol (Fig. 36).

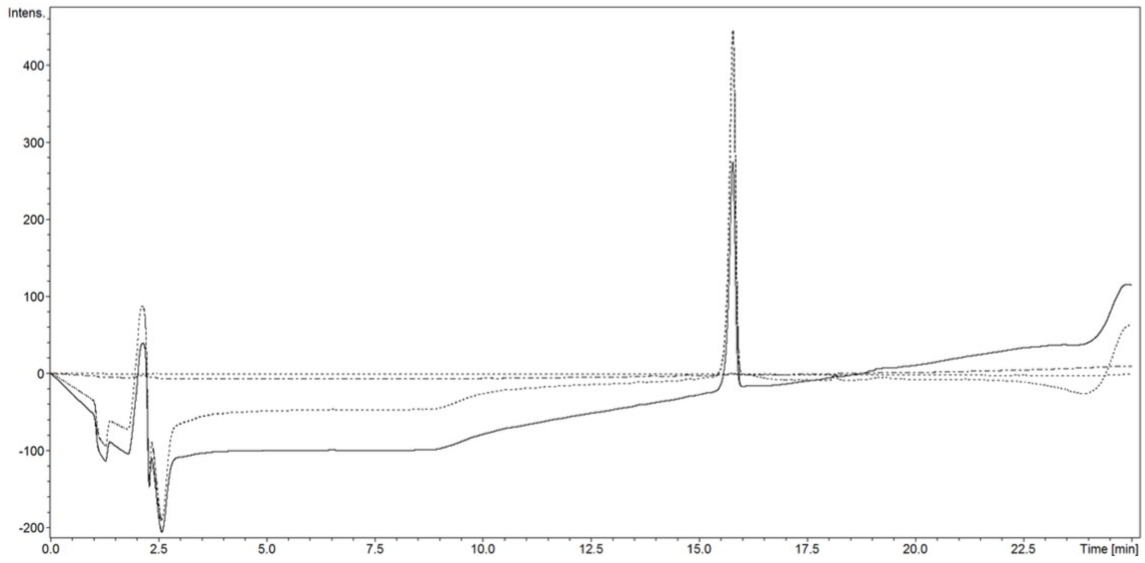

Figure S1. Chromatograph HPLC of H-GlyIleValGluGlnCysCysThrSerIle-OH (**1**).

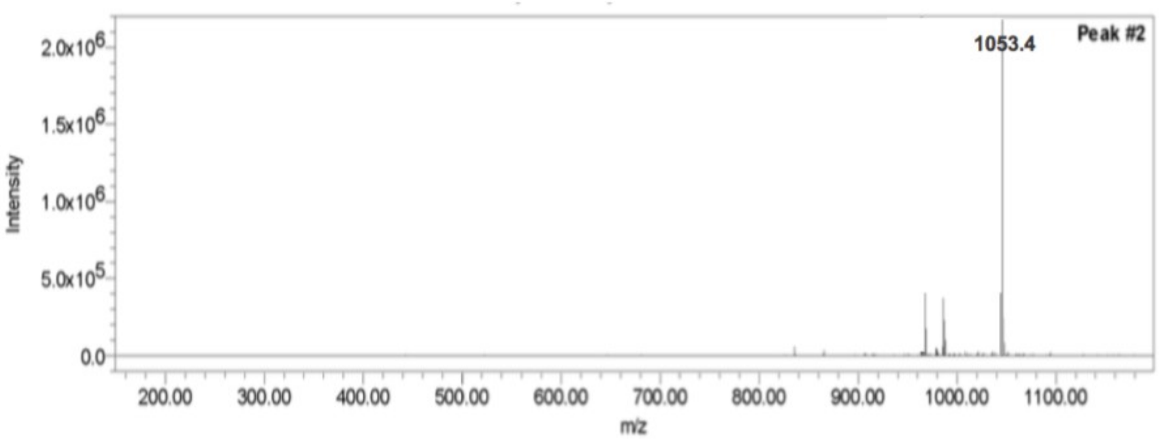

Figure S2. MS spectra of H-GlyIleValGluGlnCysCysThrSerIle-OH (**1**).

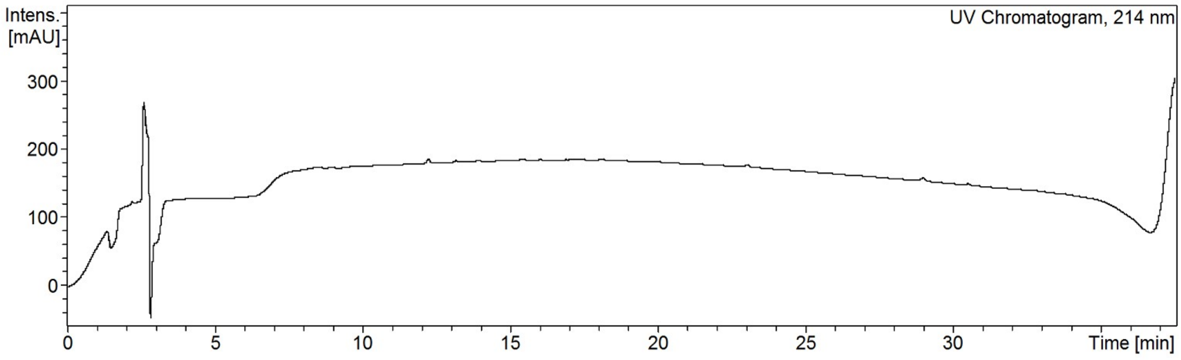

Figure S3. Chromatograph HPLC of H-GlyIleValGluGln-OH (**2**).

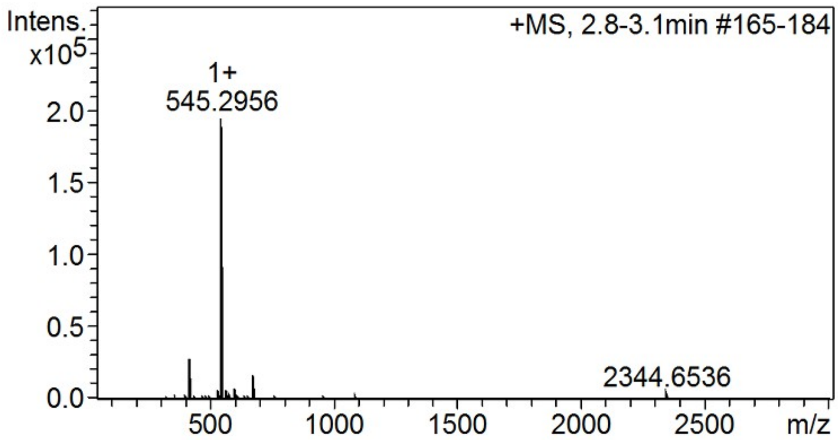

Figure S4. MS spectra of H-GlyIleValGluGln-OH (**2**).

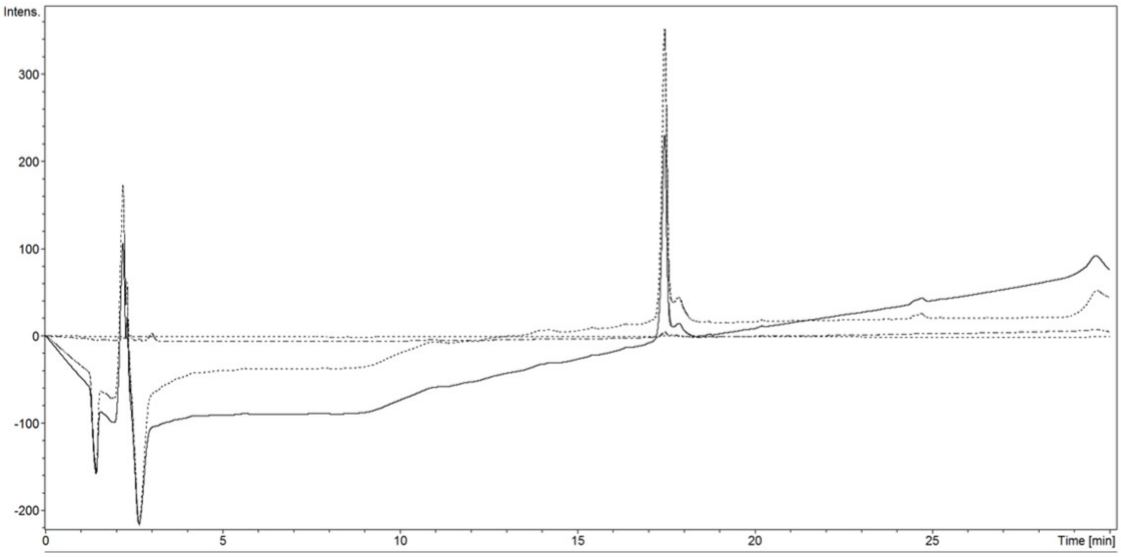

Figure S5. Chromatograph HPLC of H-CysCysThrSerIle-OH (**3**).

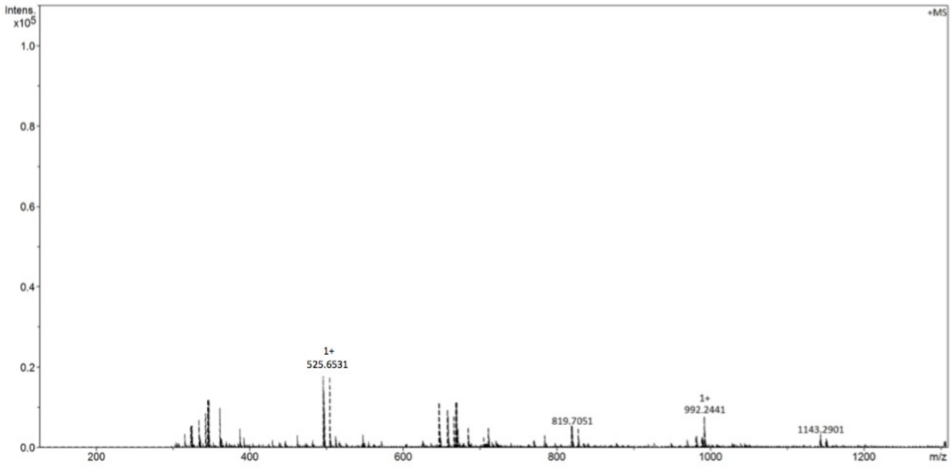

Figure S6. MS spectra of H-CysCysThrSerIle-OH (**3**).

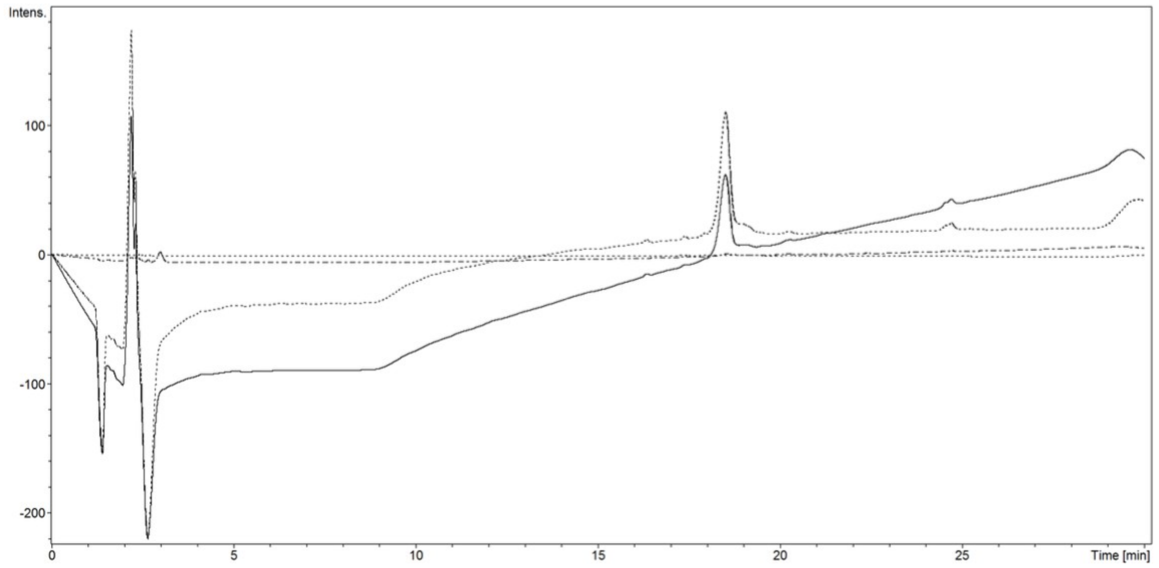

Figure S7. Chromatograph HPLC of H-CysSerLeuTyrGlnLeuGluAsnTyrCysAsn-COOH (**4**).

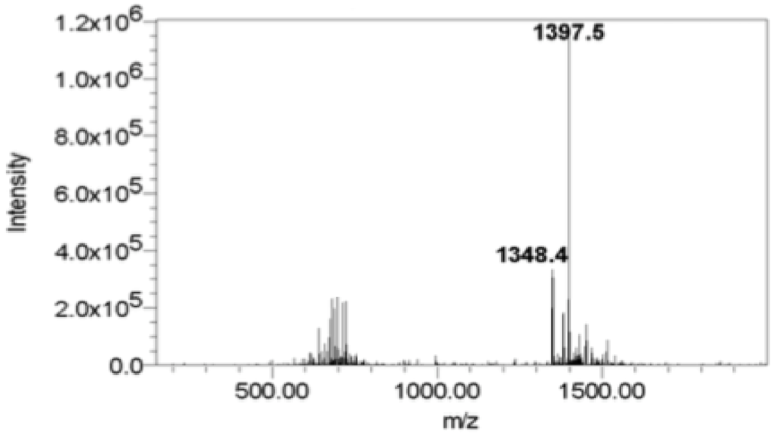

Figure S8. MS spectra of H-CysSerLeuTyrGlnLeuGluAsnTyrCysAsn-OH (**4**).

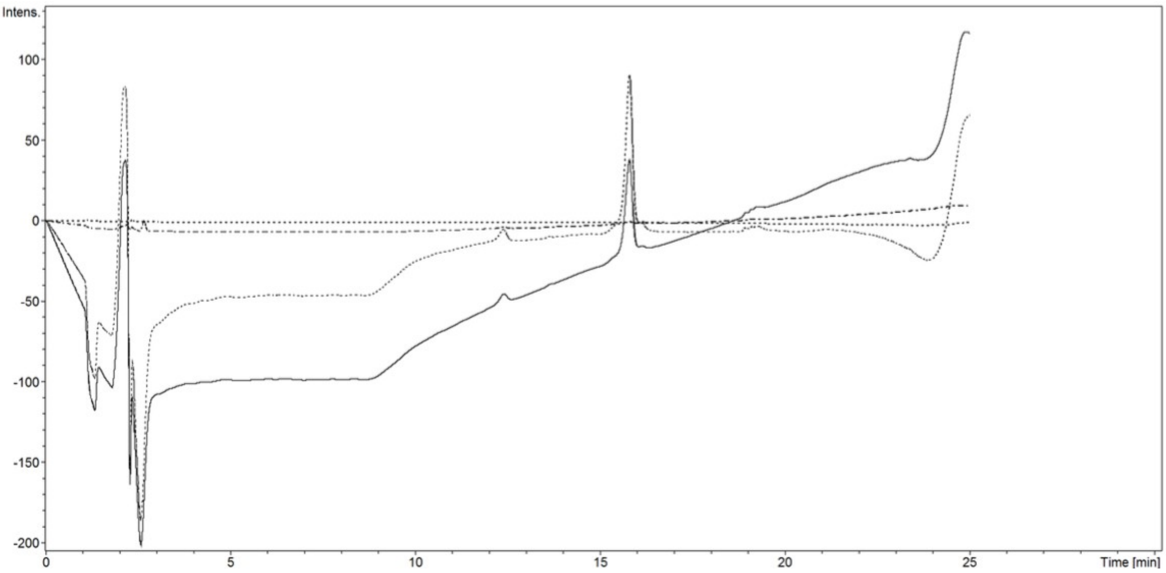

Figure S9. Chromatograph HPLC of H-CysSerLeuTyrGlnLeu-OH (**5**).

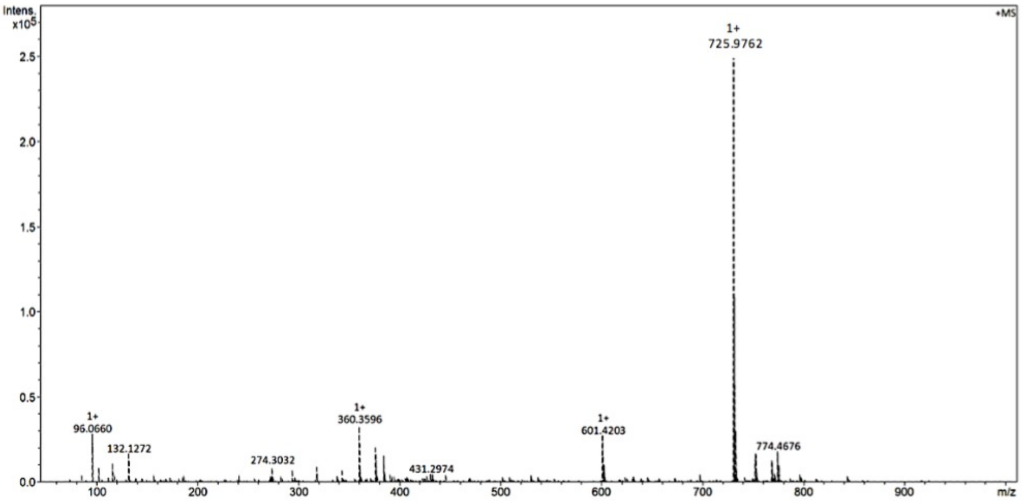

Figure S10. MS spectra of H-CysSerLeuTyrGlnLeu-OH (**5**).

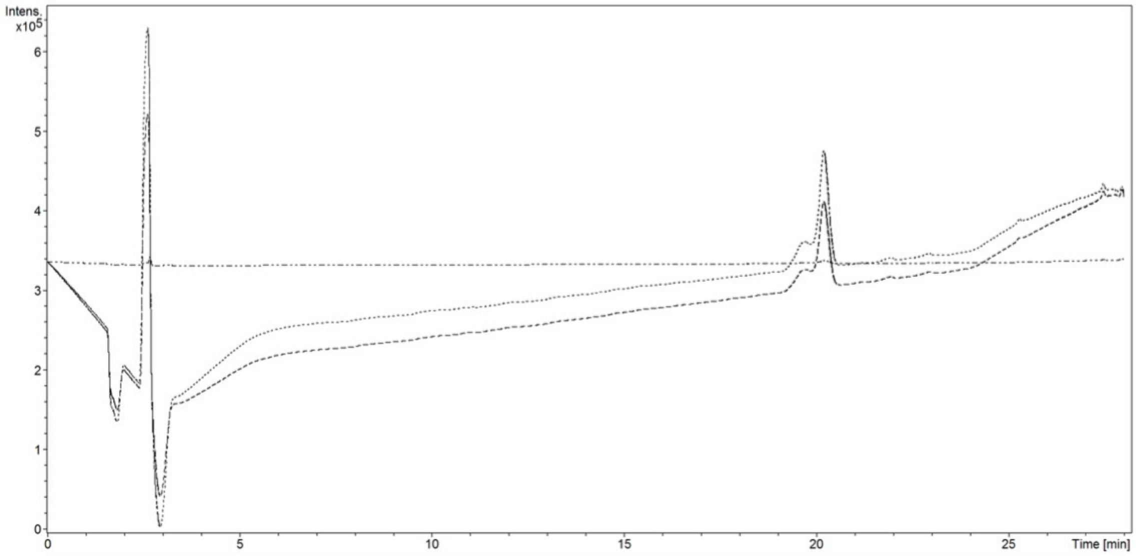

Figure S11. Chromatograph HPLC of H-GluAsnTyrCysAsn-OH (**6**).

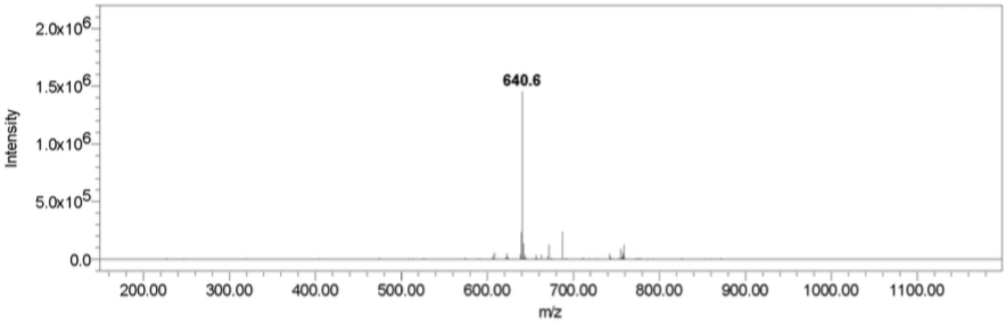

Figure S12. MS spectra of H-GluAsnTyrCysAsn-OH (**6**).

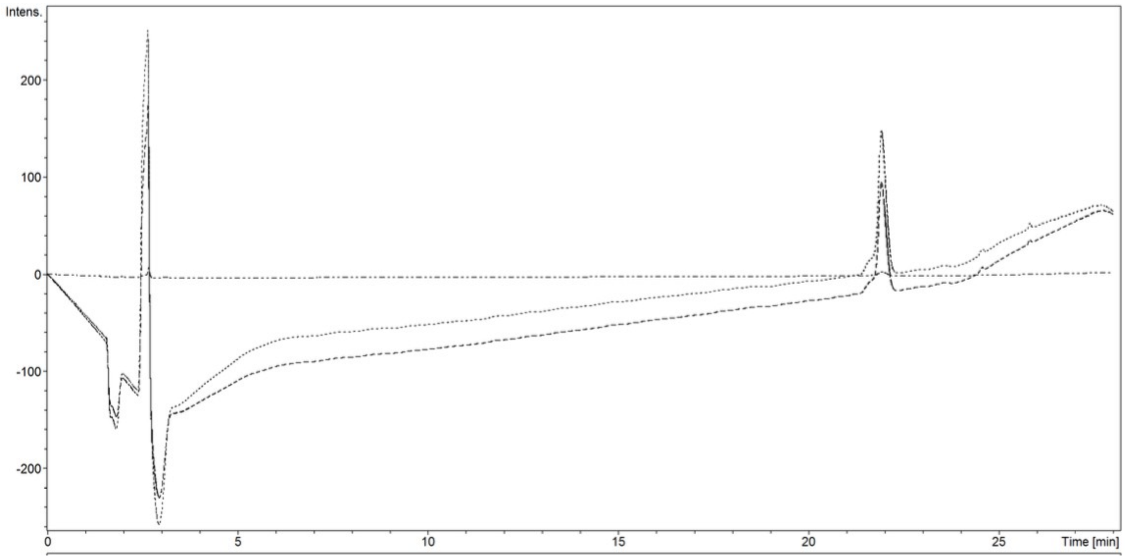

Figure S13. Chromatograph HPLC of H-PheValAsnGlnHisLeuCysGlySerHis-OH (**7**).

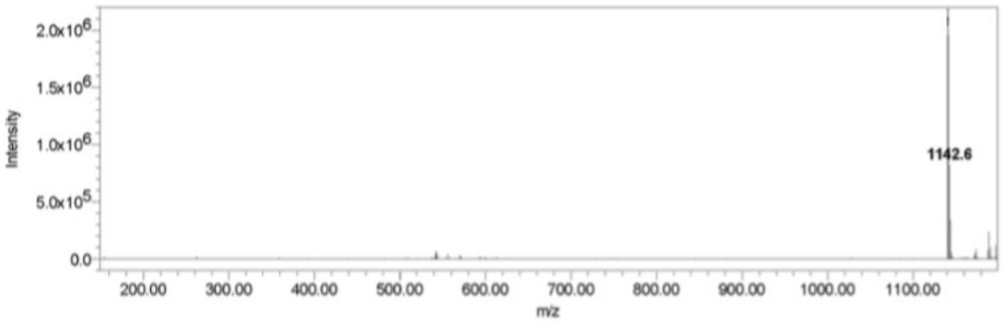

Figure S14. MS spectra of H-PheValAsnGlnHisLeuCysGlySerHis-OH (**7**).

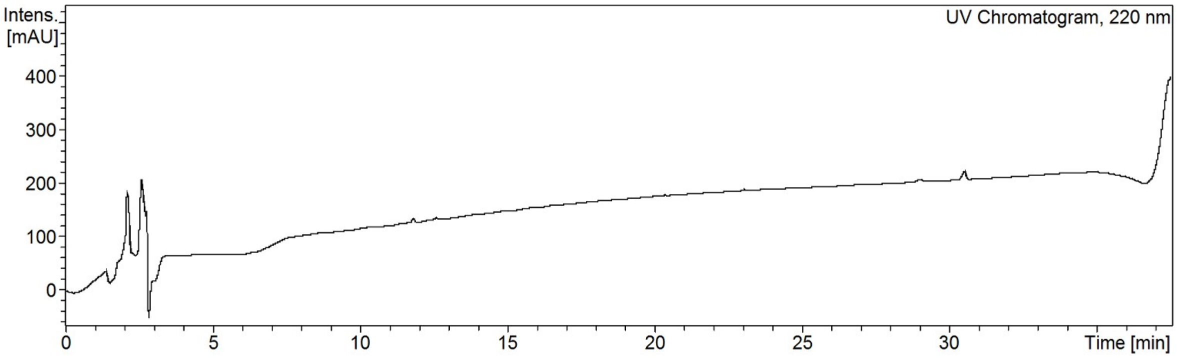

Figure S15. Chromatograph HPLC of H-PheValAsnGlnHis-OH (**8**).

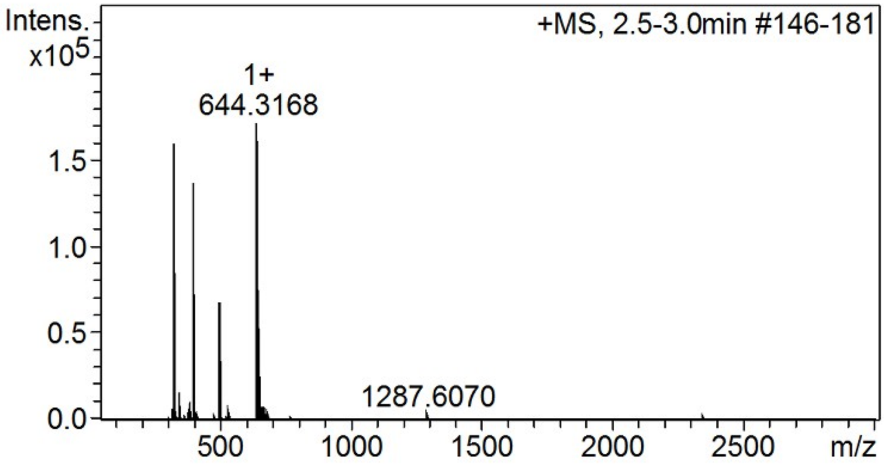

Figure S16. MS spectra of H-PheValAsnGlnHis-OH (**8**).

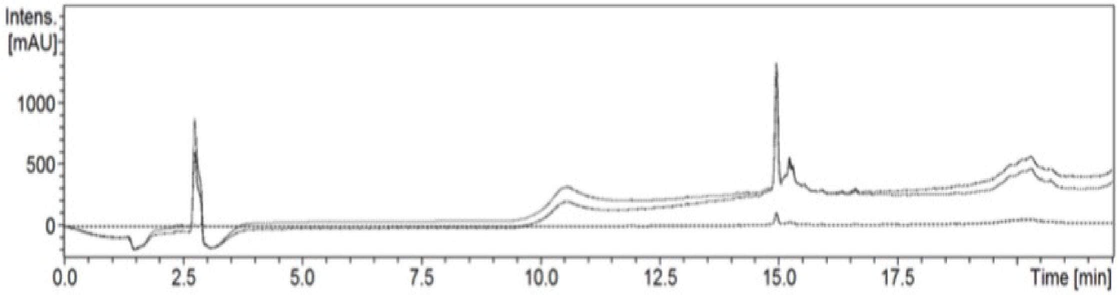

Figure S17. Chromatograph HPLC of H-LeuCysGlySerHis-OH (**9**).

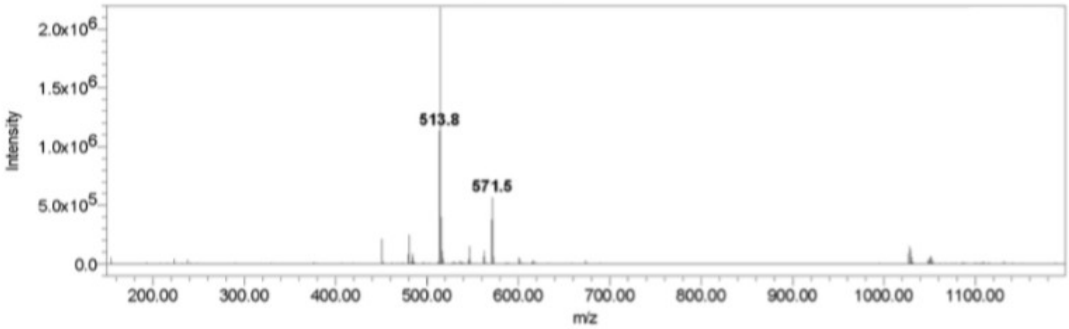

Figure S18. MS spectra of H-LeuCysGlySerHis-OH (**9**).

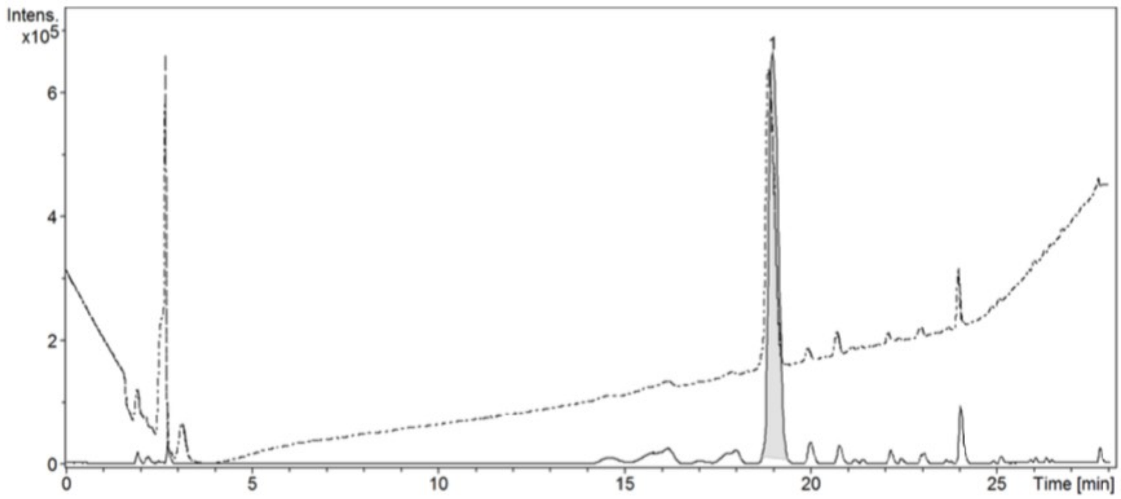

Figure S19. Chromatograph HPLC of H-LeuValGluAlaLeuTyrLeuValCysGly-OH (**10**).

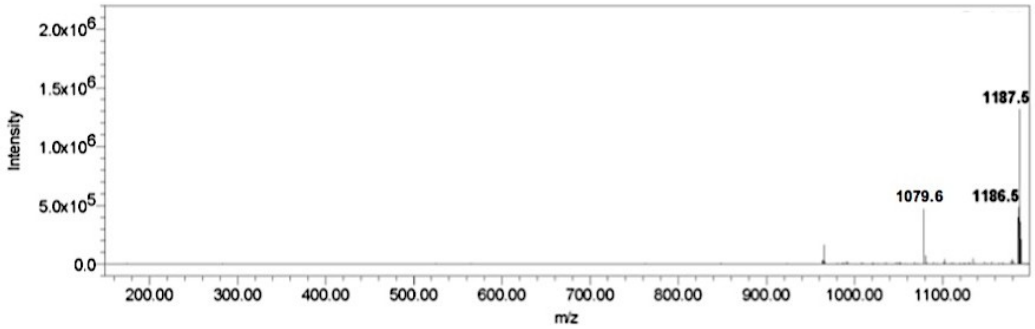

Figure S20. MS spectra of H-LeuValGluAlaLeuTyrLeuValCysGly-OH (**10**).

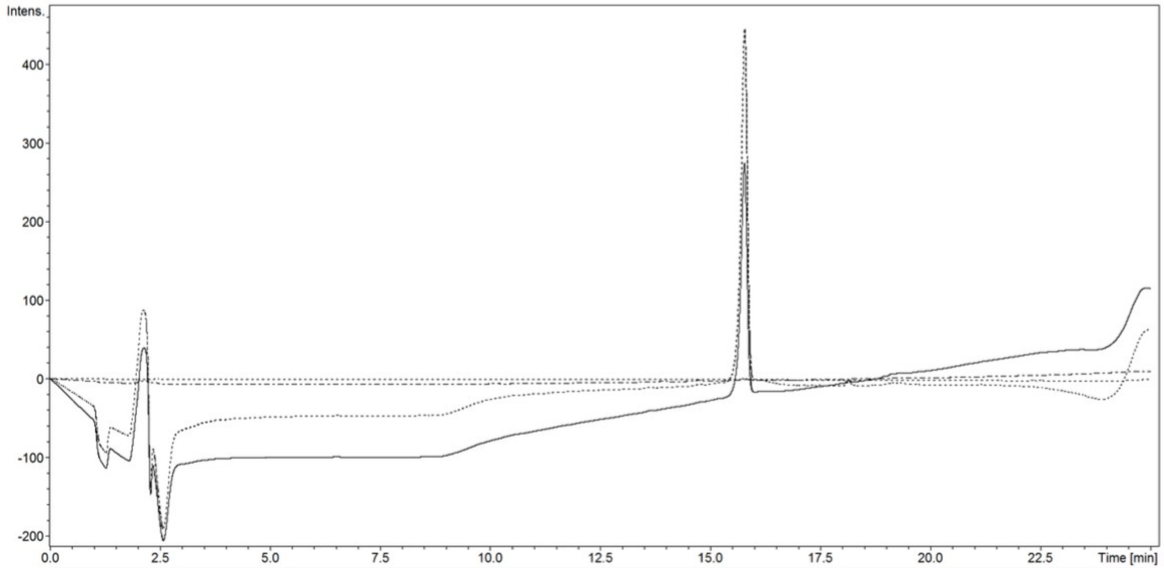

Figure S21. Chromatograph HPLC of H-LeuValGluAlaLeu-OH (**11**).

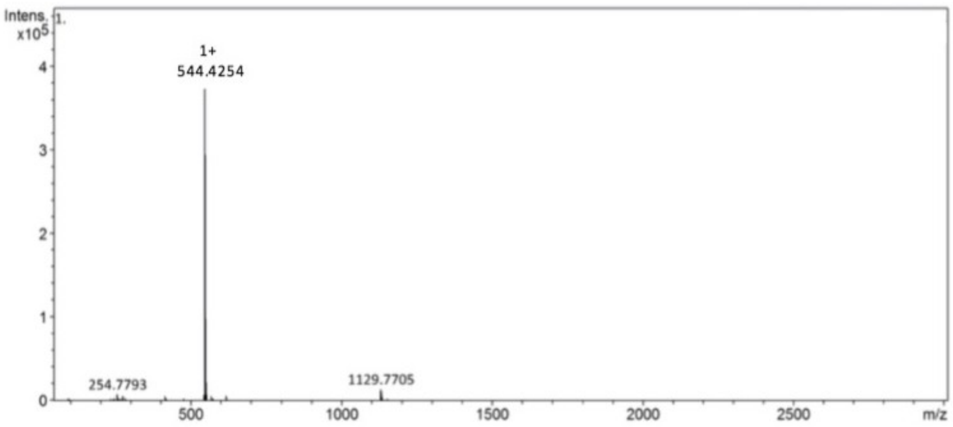

Figure S22. MS spectra of H-LeuValGluAlaLeu-OH (**11**).

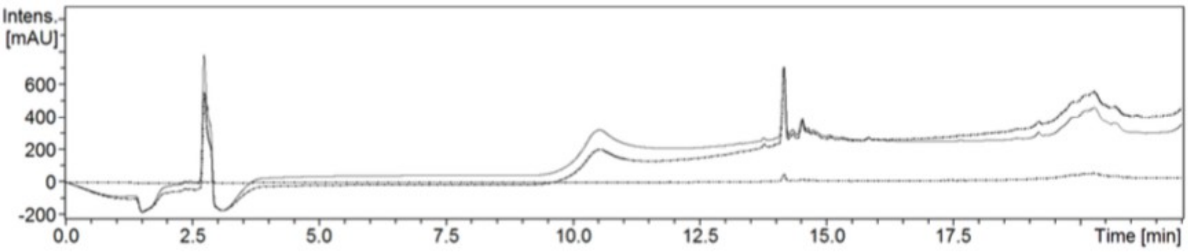

Figure S23. Chromatograph HPLC of H-TyrLeuValCysGly-OH (**12**).

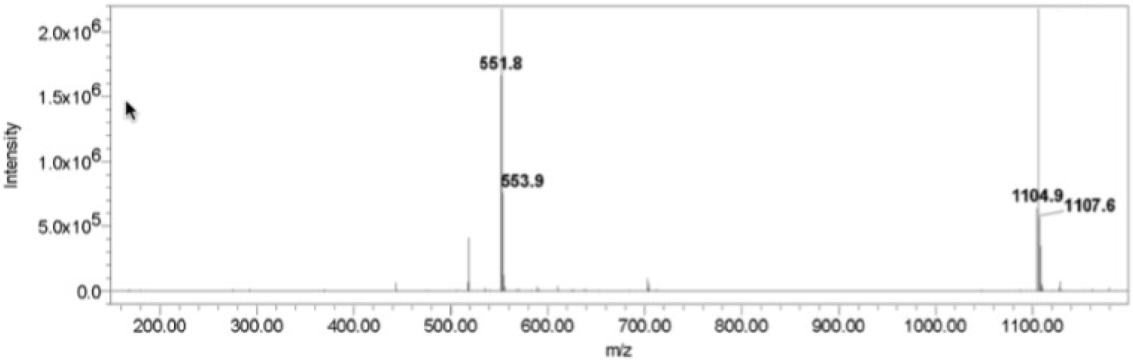

Figure S24. MS spectra of H-TyrLeuValCysGly-OH (**12**).

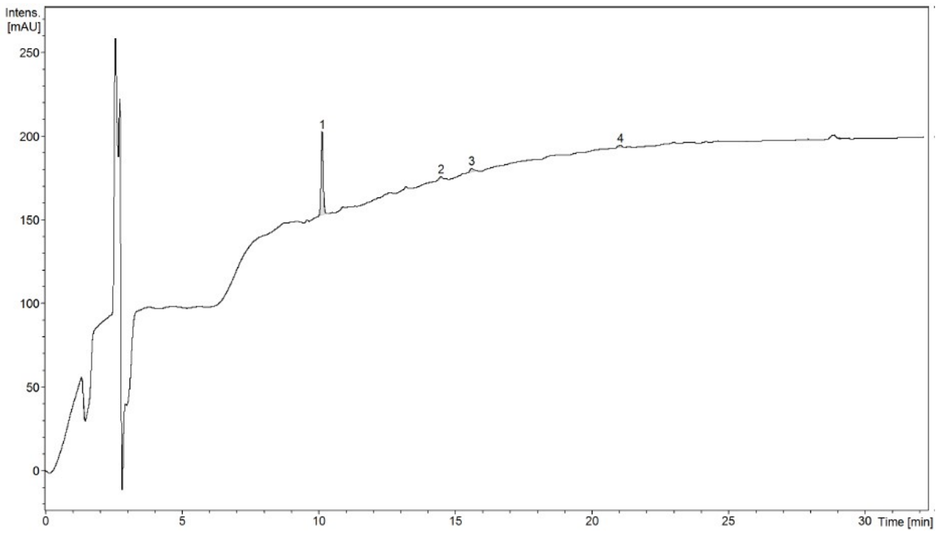

Figure S25. Chromatograph HPLC of H-GluArgGlyPhePheTyrThrProLysThr-OH (**13**).

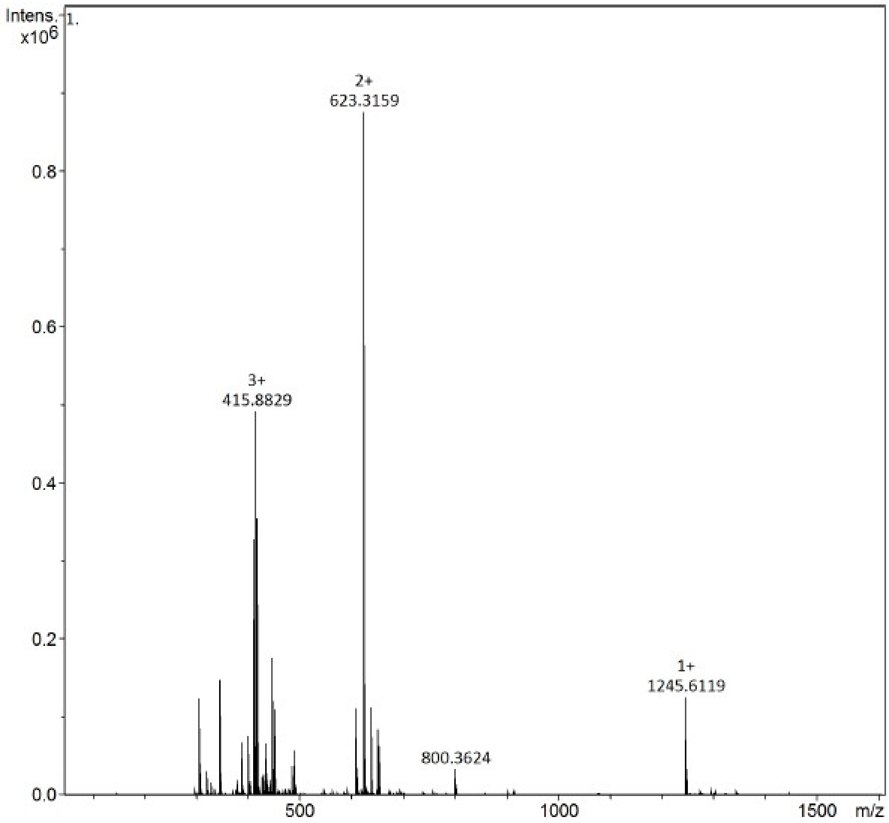

Figure S26. MS spectra of H-GluArgGlyPhePheTyrThrProLysThr-OH (**13**).

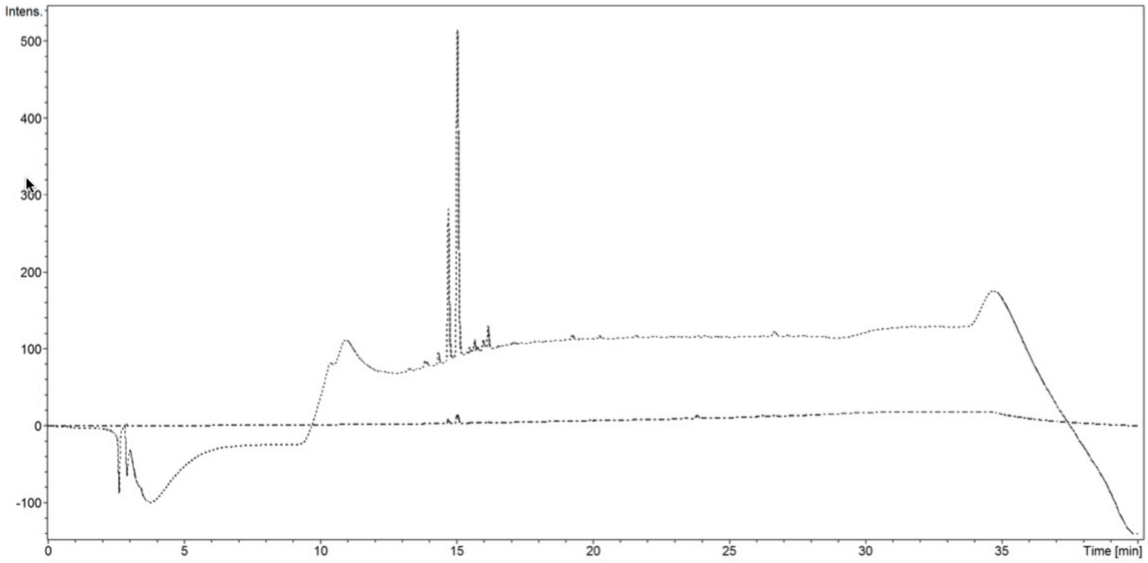

Figure S27. Chromatograph HPLC of H-GluArgGlyPhePhe-OH (**14**).

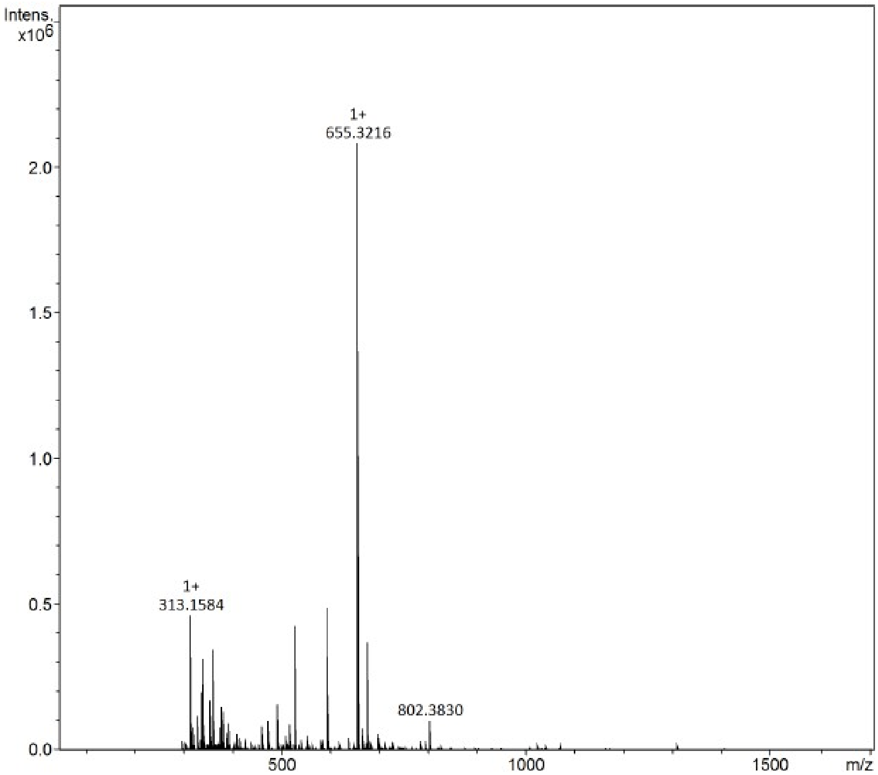

Figure S28. MS spectra of H-GluArgGlyPhePhe-COOH (**14**).

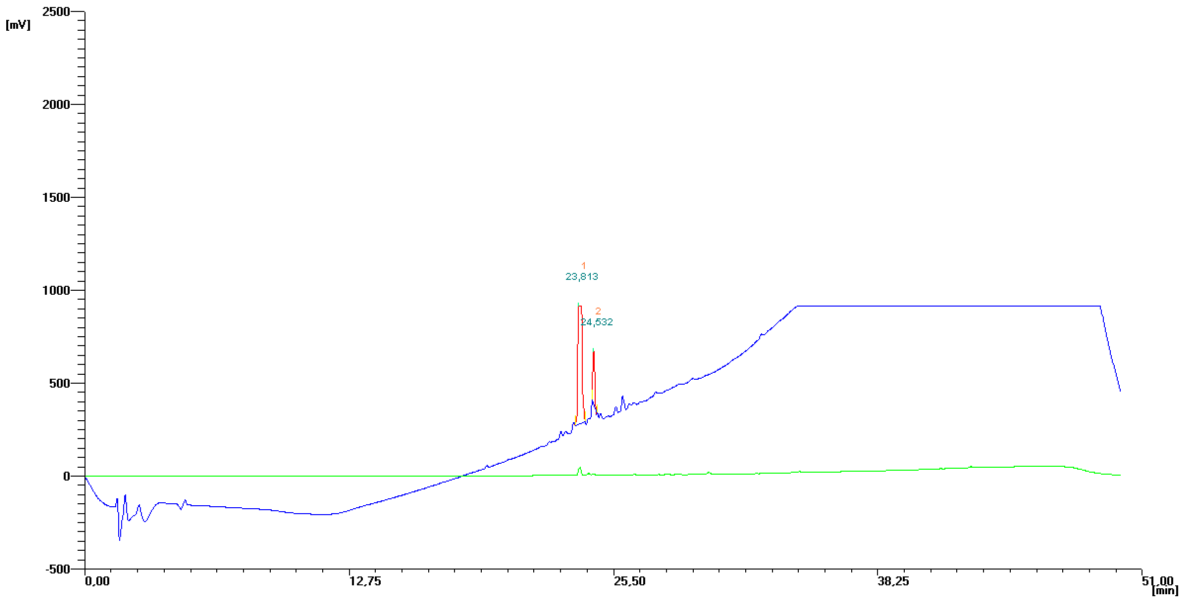

Figure S29. Chromatograph HPLC of H-TyrThrProLysThr-OH (**15**).

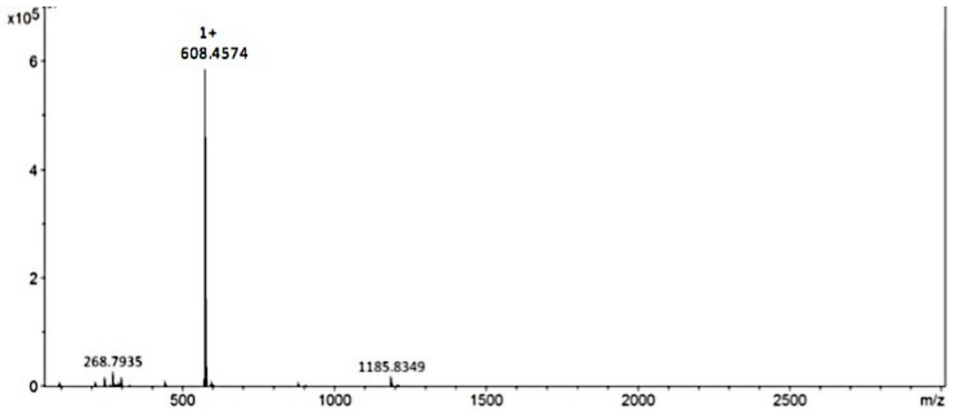

Figure S30. MS spectra of H-TyrThrProLysThr-OH (**15**).

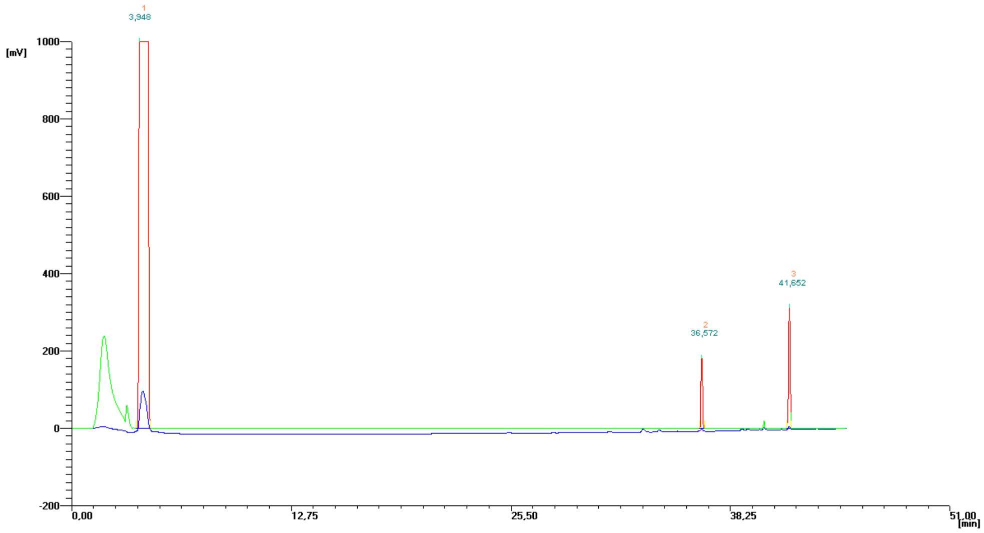

Figure S31. Chromatograph HPLC of H-LeuTyrGlnLeuGluAsnTyr-OH (**16**).

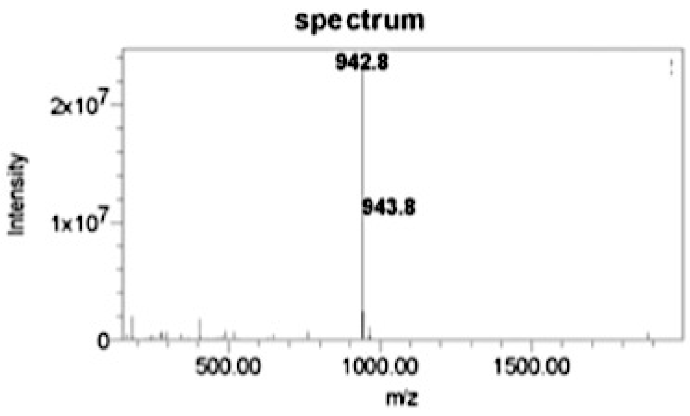

Figure S32. MS spectra of H-LeuTyrGlnLeuGluAsnTyr-OH (**16**).

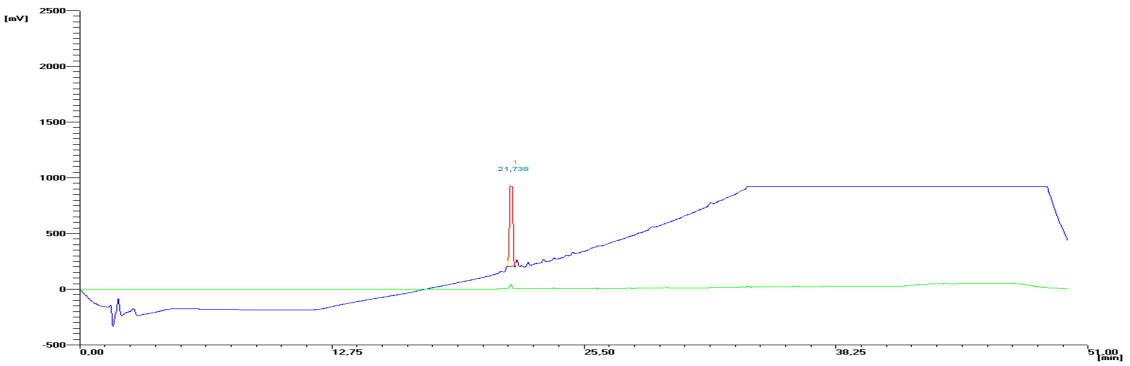

Figure S33. Chromatograph HPLC of H-ValGluAlaLeuTyrLeu-OH (**17**).

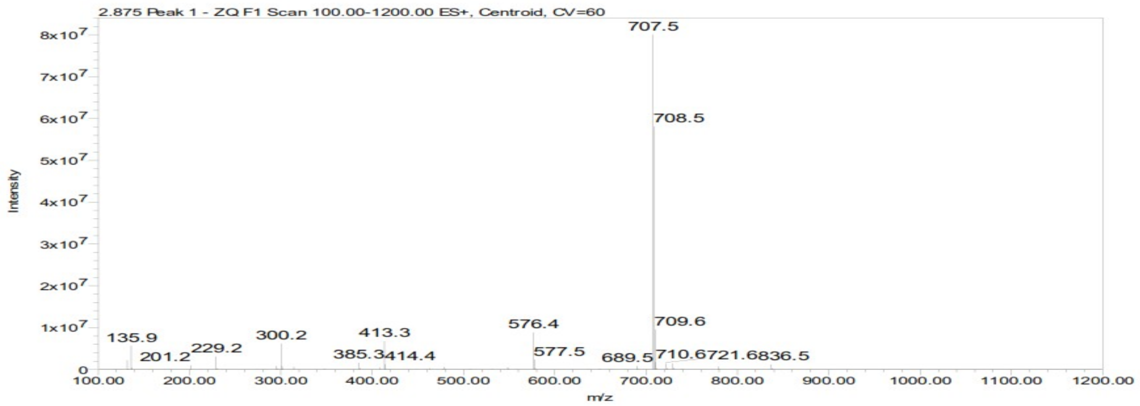

Figure S34. MS spectra of H-ValGluAlaLeuTyrLeu-OH (**17**).

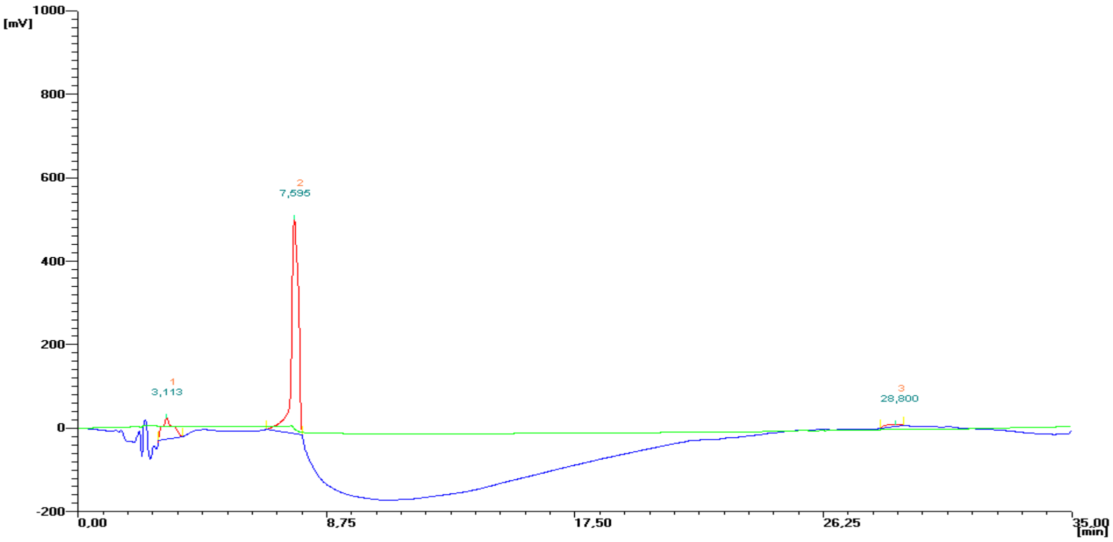

Figure S35. Chromatograph HPLC of H-ArgGlyPhePheTyrThr-OH (**18**).

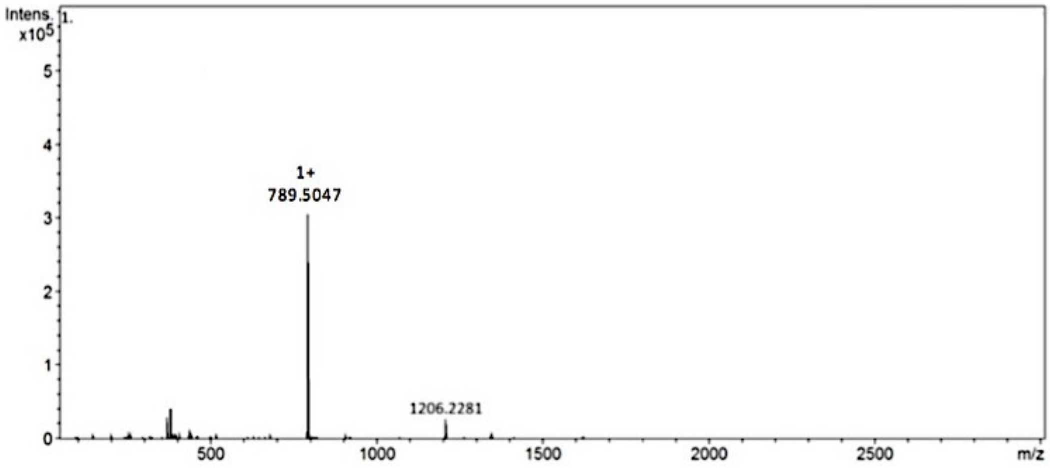

Figure S36. MS spectra of H-ArgGlyPhePheTyrThr-OH (**18**).

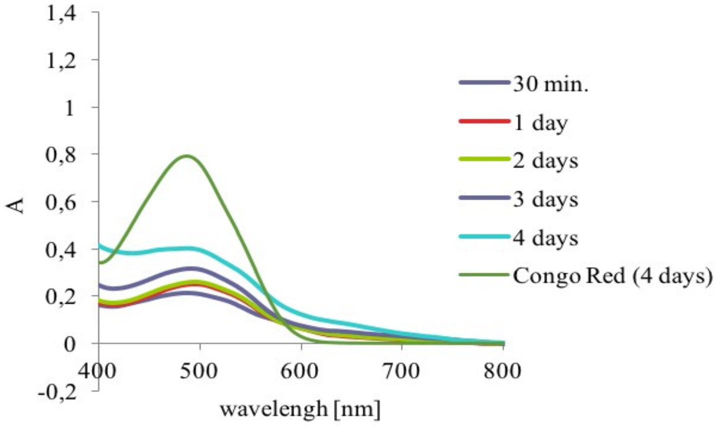

Figure S37. UV spectra of H-GlyIleValGluGlnCysCysThrSerIle-OH (**1**).

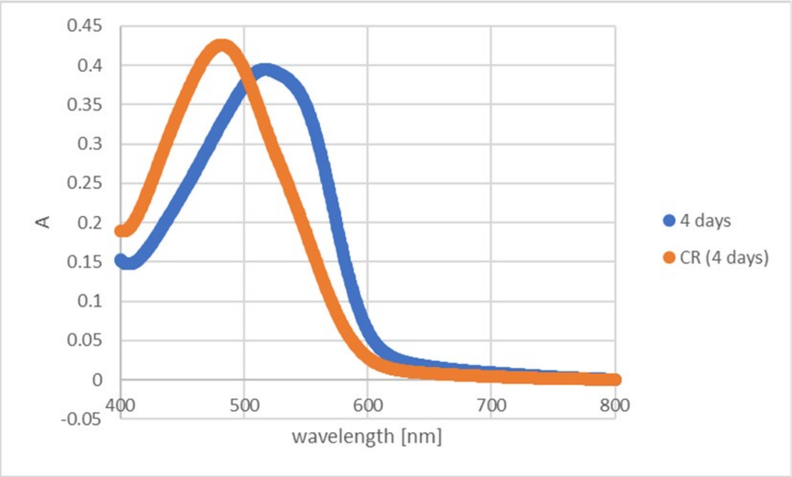

Figure S38. UV spectra of H-GlyIleValGluGln-OH (**2**).

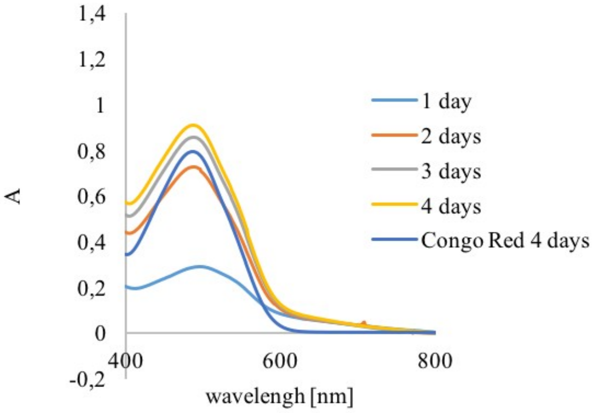

Figure S39. UV spectra of H-CysCysThrSerIle-OH (**3**).

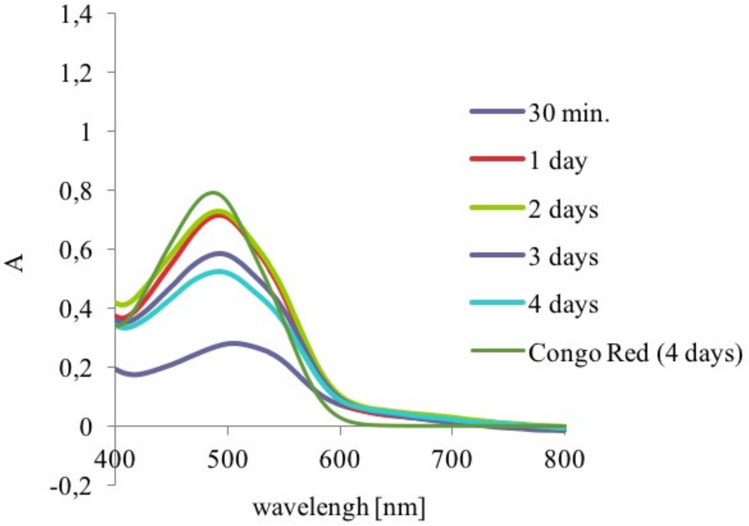

Figure S40. UV spectra of H-CysSerLeuTyrGlnLeuGluAsnTyrCysAsn-OH (**4**).

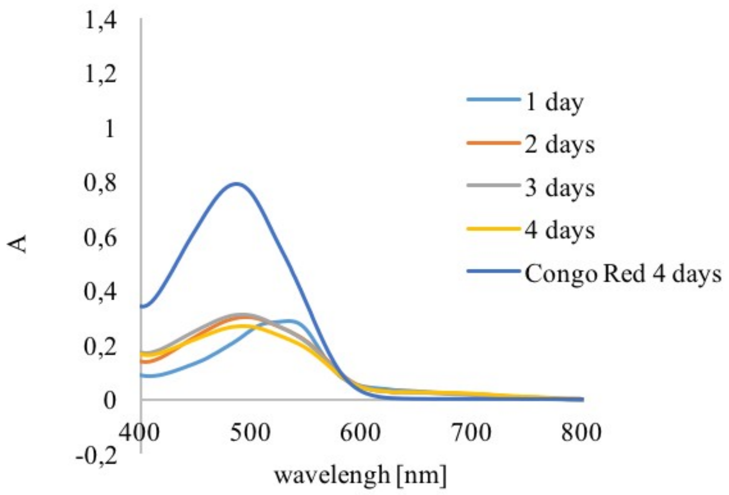

Figure S41. UV spectra of H-CysSerLeuTyrGlnLeu-OH (**5**).

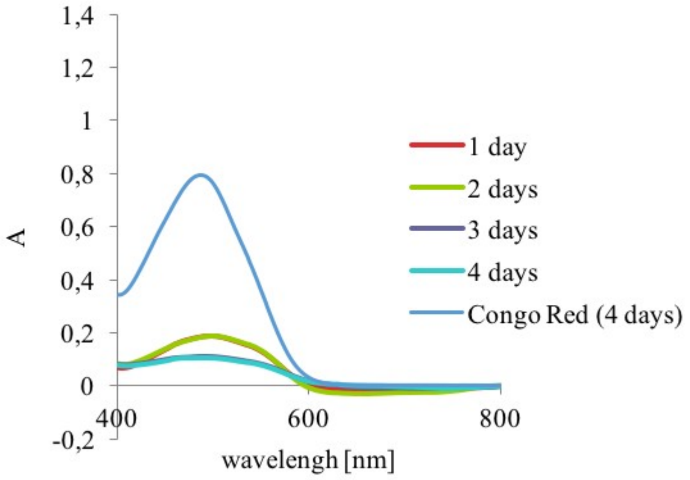

Figure S42. UV spectra of H-GluAsnTyrCysAsn-OH (**6**).

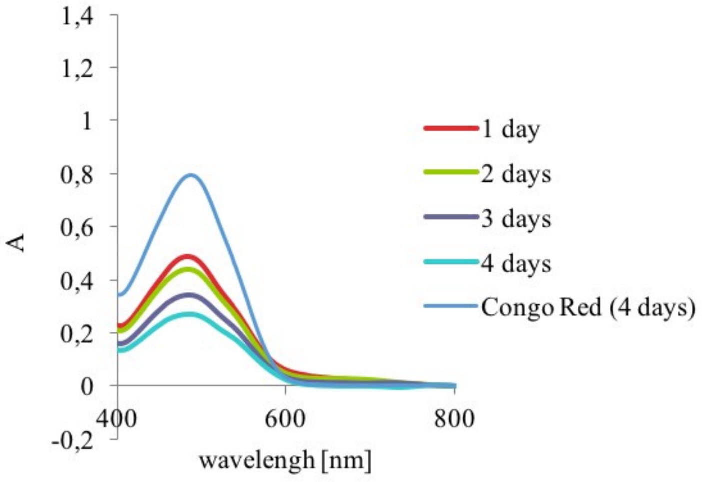

Figure S43. UV spectra of H-PheValAsnGlnHisLeuCysGlySerHis-OH (**7**).

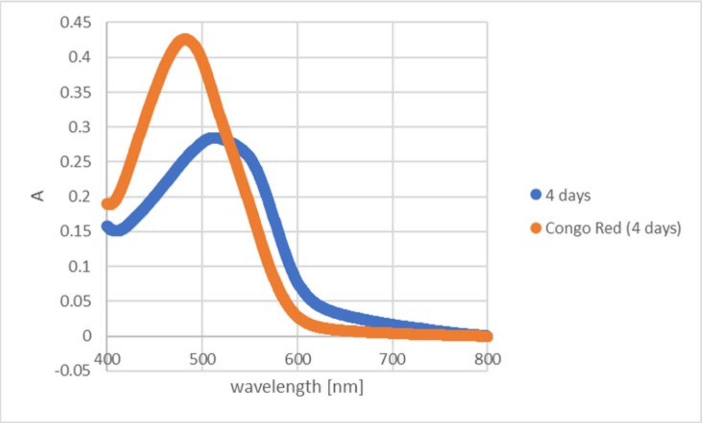

Figure S44. UV spectra of H-PheValAsnGlnHis-OH (**8**).

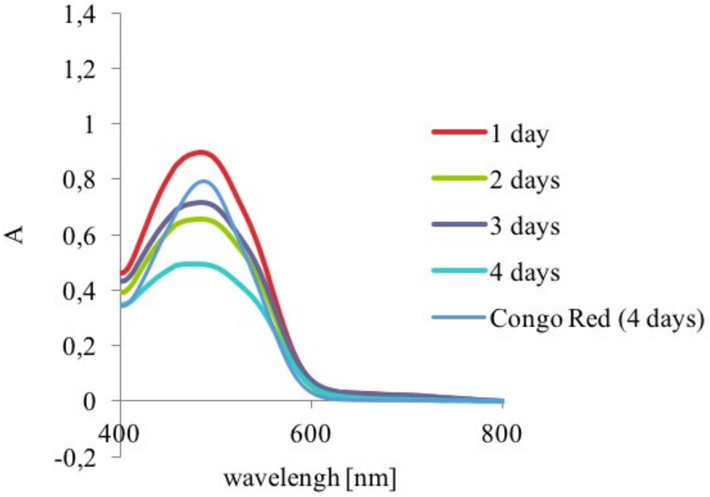

Figure S45. UV spectra of H-LeuCysGlySerHis-OH (**9**).

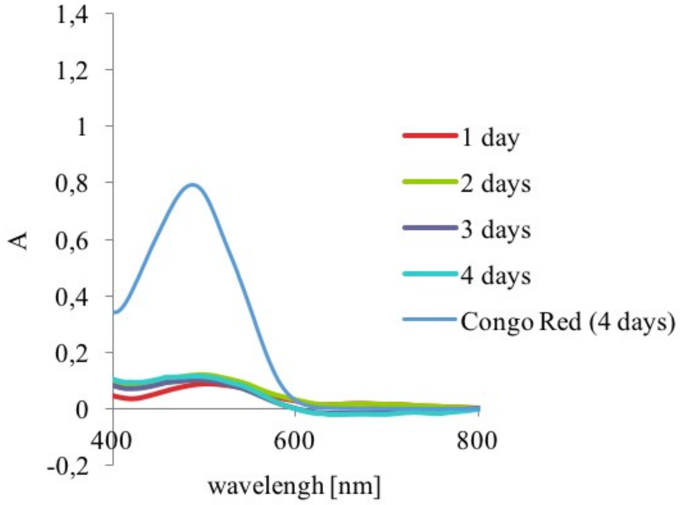

Figure S46. UV spectra of H-LeuValGluAlaLeuTyrLeuValCysGly-OH (**10**).

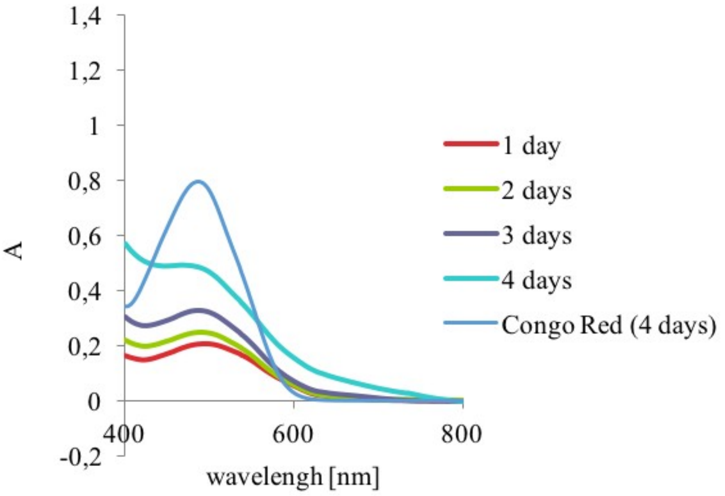

Figure S47. UV spectra of H-LeuValGluAlaLeu-OH (**11**).

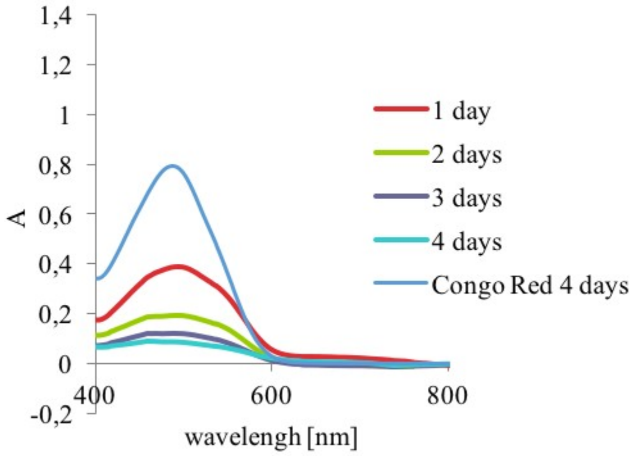

Figure S48. UV spectra of H-TyrLeuValCysGly-OH (**12**).

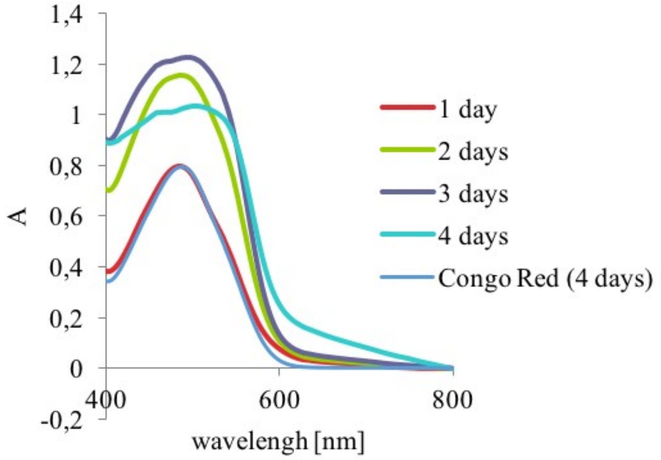

Figure S49. UV spectra of H-GluArgGlyPhePheTyrThrProLysThr-OH (**13**).

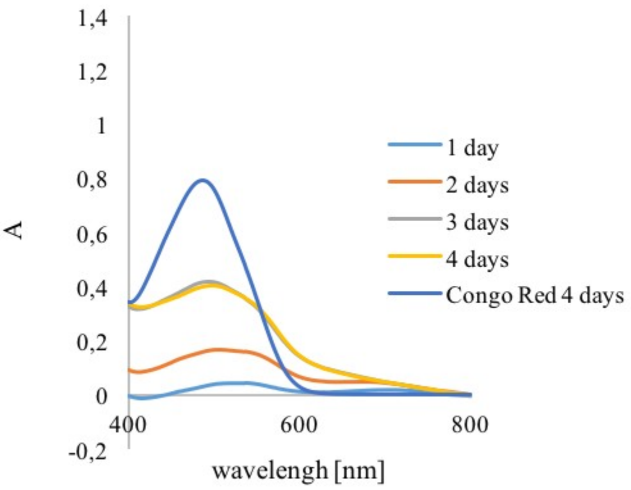

Figure S50. UV spectra of H-GluArgGlyPhePhe-COOH (**14**).

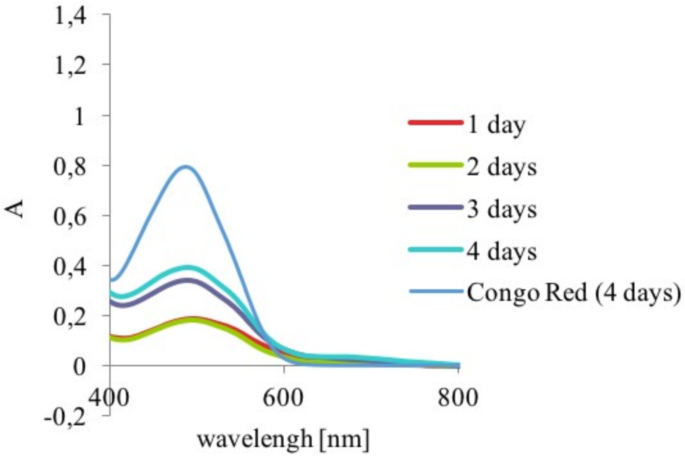

Figure S51. UV spectra of H-TyrThrProLysThr-OH (**15**).

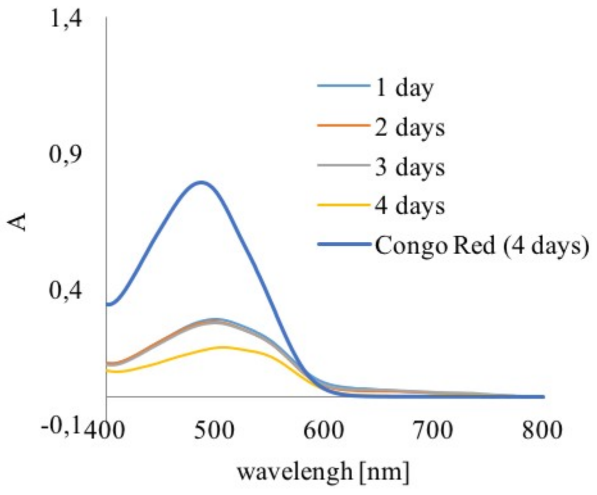

Figure S52. UV spectra of H-LeuTyrGlnLeuGluAsnTyr-OH (**16**).

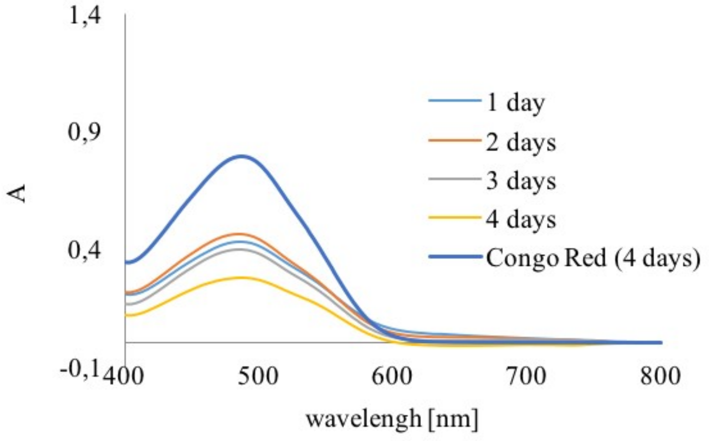

Figure S53. UV spectra of H-ValGluAlaLeuTyrLeu-OH (**17**).

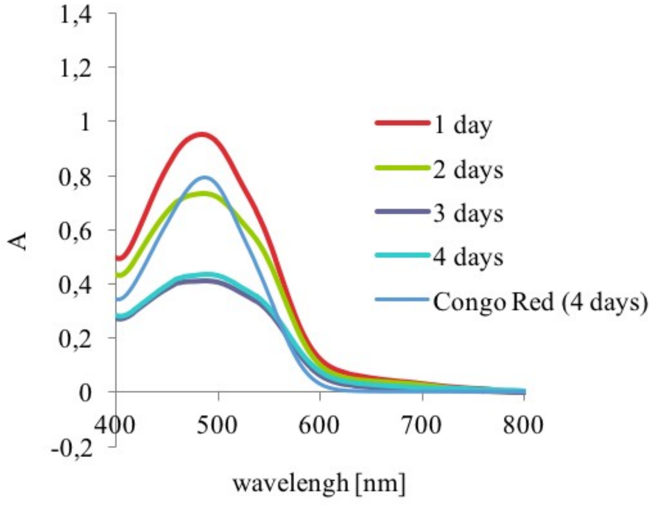

Figure S54. UV spectra of H-ArgGlyPhePheTyrThr-OH (**18**).

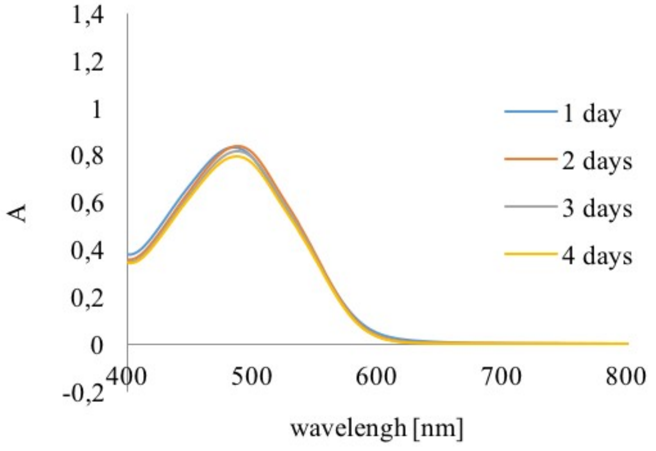

Figure S55. UV spectra of Congo Red.

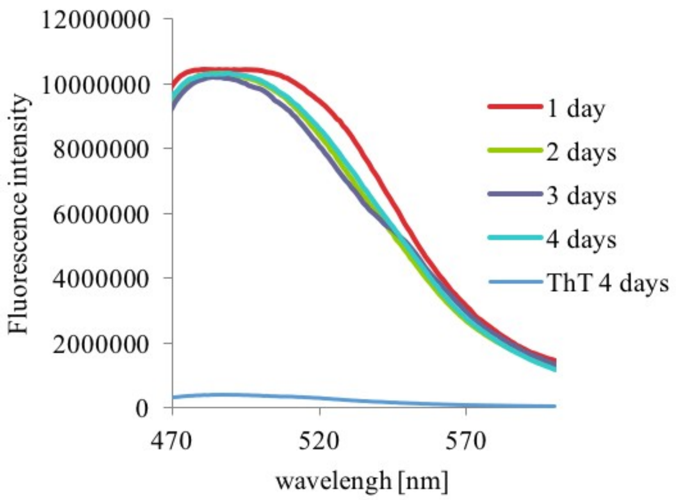

Figure S56. Fluorescence spectra of H-GlyIleValGluGlnCysCysThrSerIle-OH (**1**).

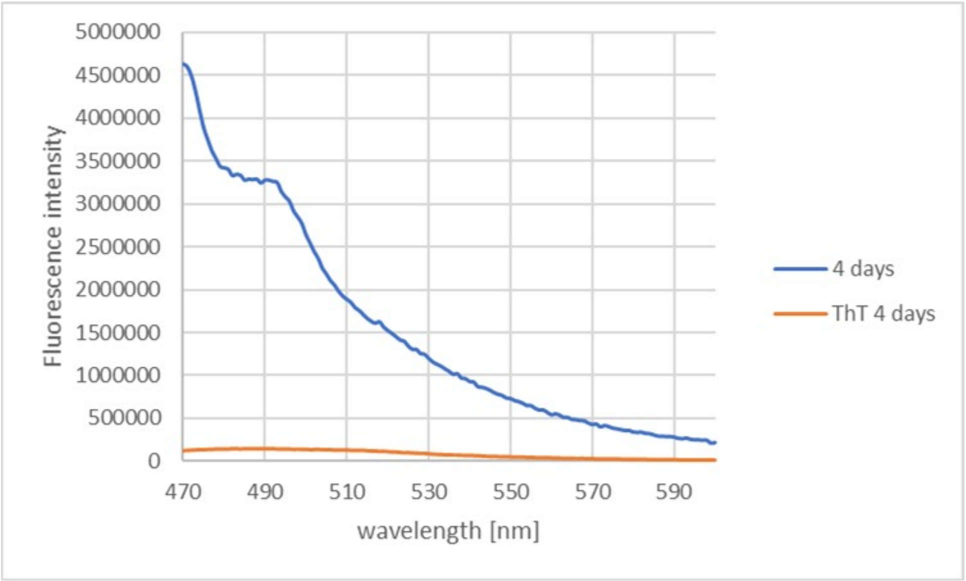

Figure S57. Fluorescence spectra of H-GlyIleValGluGln-OH (**2**).

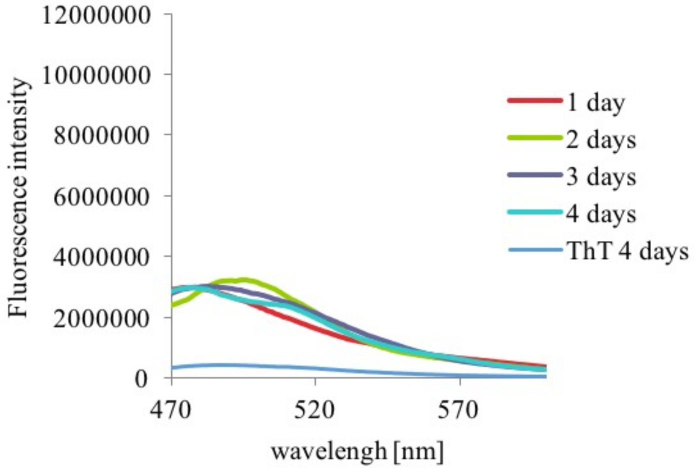

Figure S58. Fluorescence spectra of H-CysCysThrSerIle-OH (**3**).

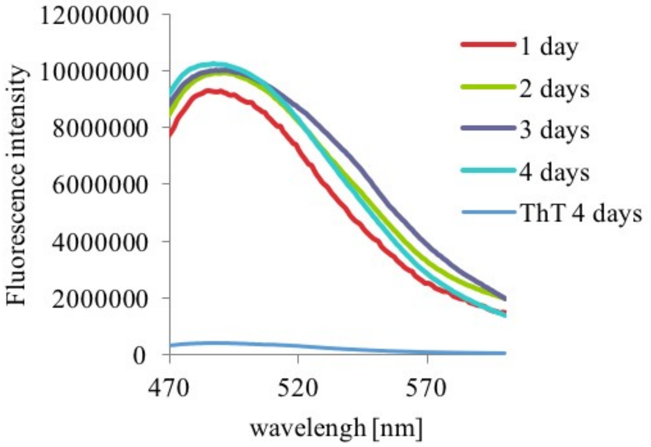

Figure S59. Fluorescence spectra of H-CysSerLeuTyrGlnLeuGluAsnTyrCysAsn-OH (**4**).

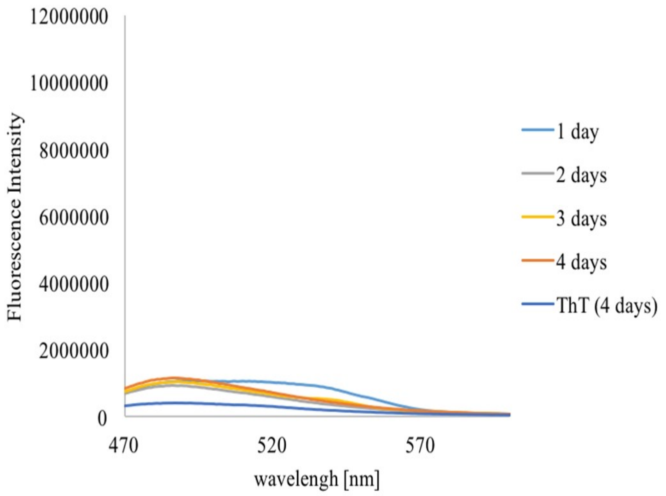

Figure S60. Fluorescence spectra of H-CysSerLeuTyrGlnLeu-OH (**5**).

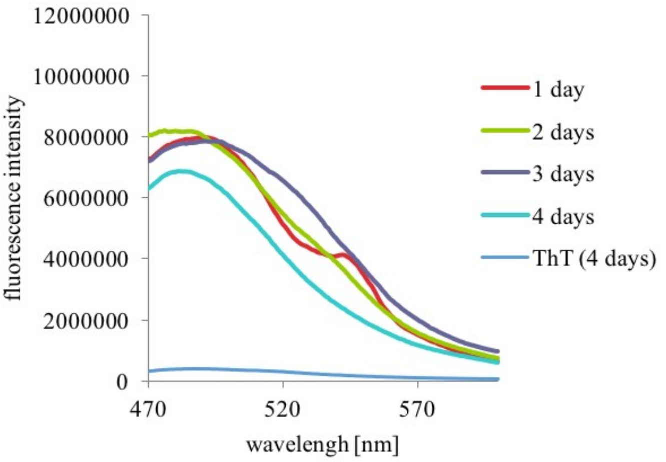

Figure S61. Fluorescence spectra of H-GluAsnTyrCysAsn-OH (**6**).

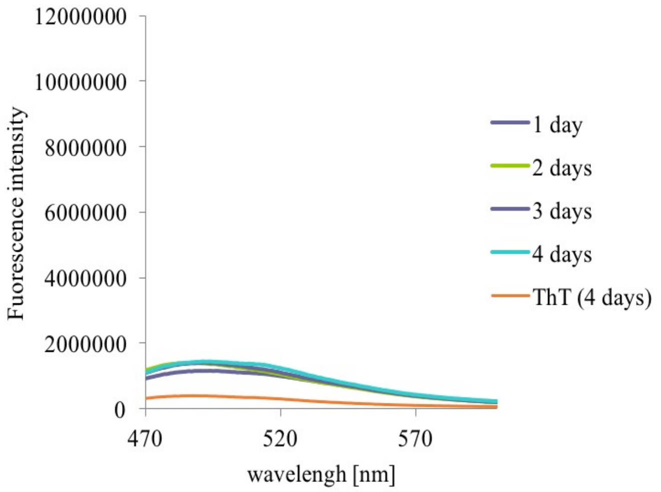

Figure S62. Fluorescence spectra of H-PheValAsnGlnHisLeuCysGlySerHis-OH (**7**).

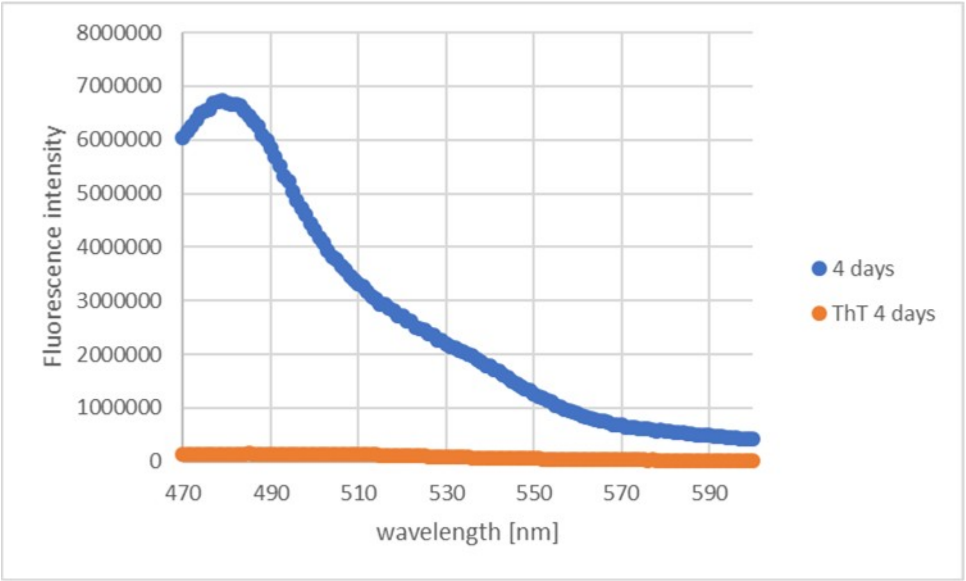

Figure S63. Fluorescence spectra of H-PheValAsnGlnHis-OH (**8**).

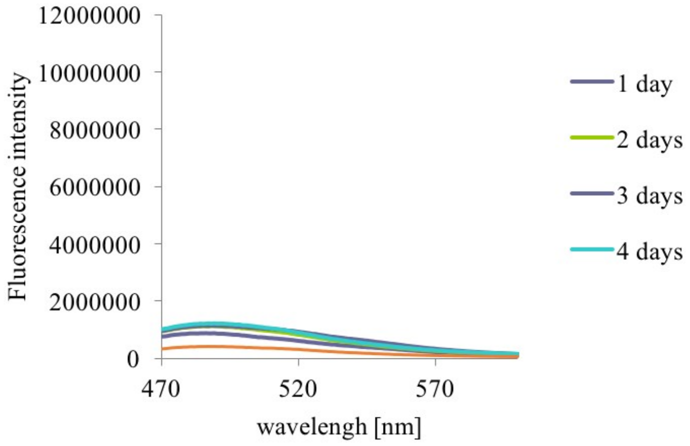

Figure S64. Fluorescence spectra of H-LeuCysGlySerHis-OH (**9**).

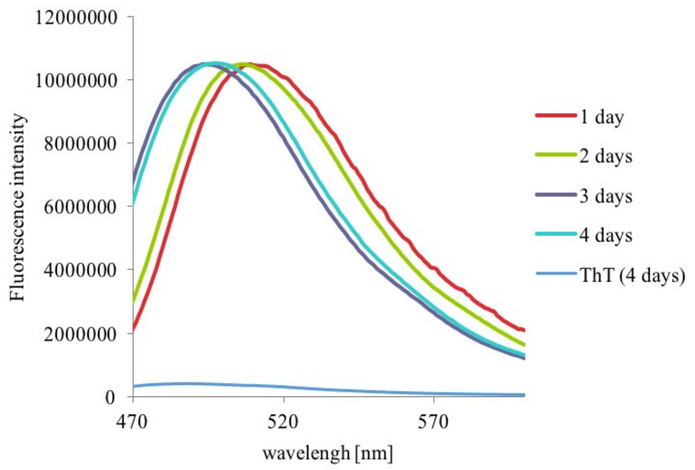

Figure S65. Fluorescence spectra of H-LeuValGluAlaLeuTyrLeuValCysGly-OH (**10**).

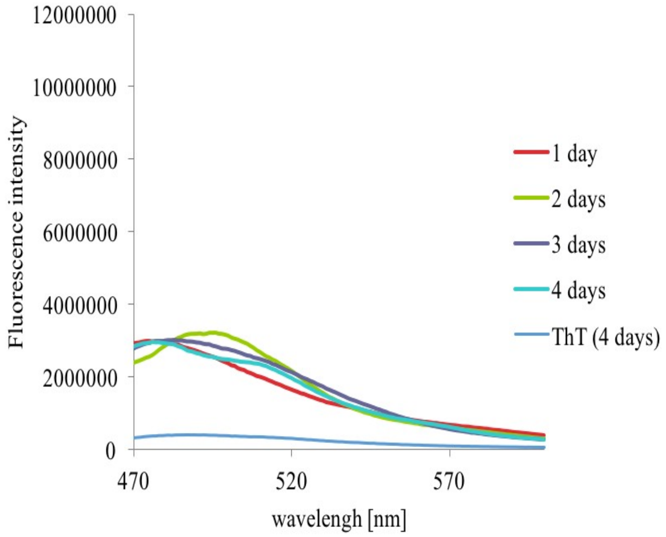

Figure S66. Fluorescence spectra of H-LeuValGluAlaLeu-OH (**11**).

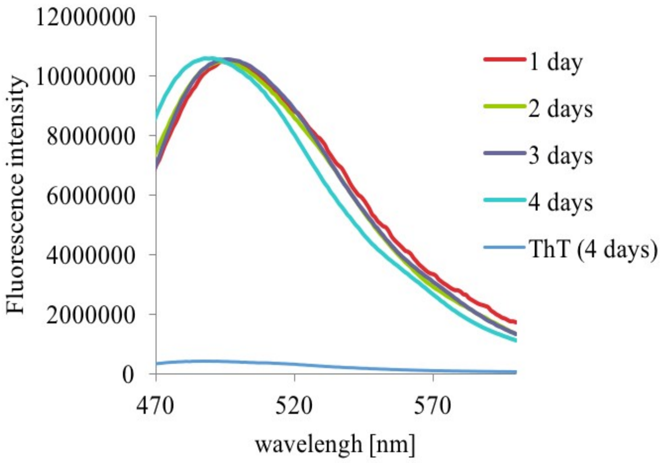

Figure S67. Fluorescence spectra of H-TyrLeuValCysGly-OH (**12**).

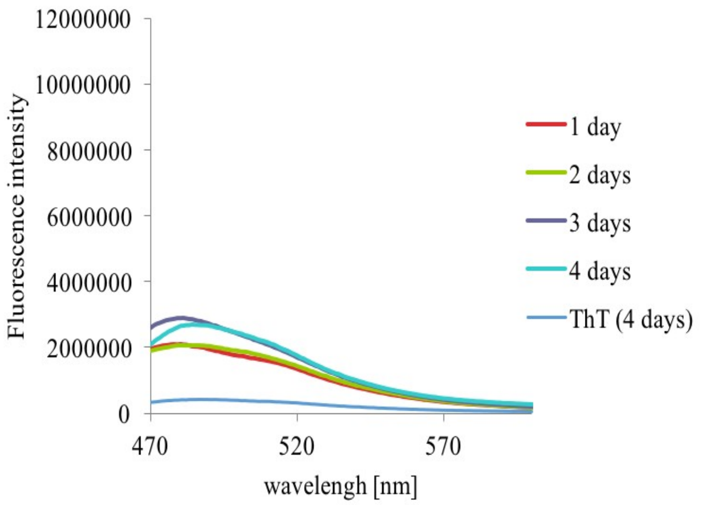

Figure S68. Fluorescence spectra of H-GluArgGlyPhePheTyrThrProLysThr-OH (**13**).

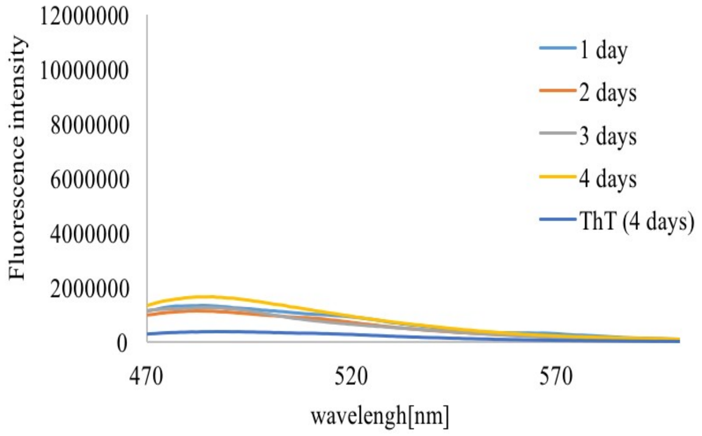

Figure S69. Fluorescence spectra of H-GluArgGlyPhePhe-OH (**14**).

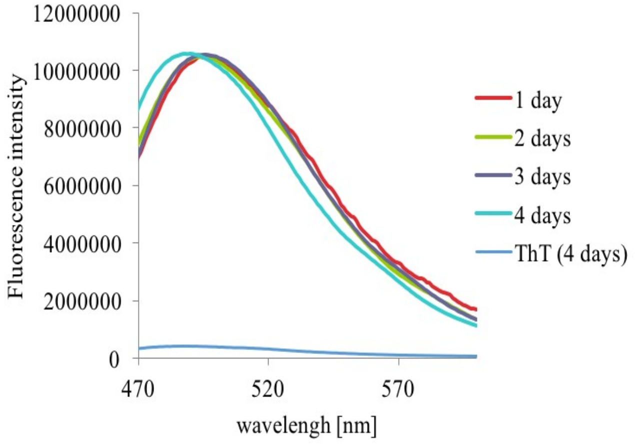

Figure S70. Fluorescence spectra of H-TyrThrProLysThr-OH (**15**).

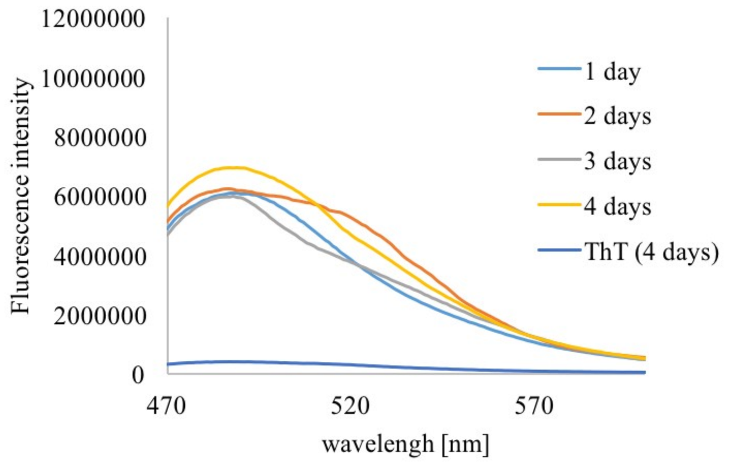

Figure S71. Fluorescence spectra of H-LeuTyrGlnLeuGluAsnTyr-COOH (**16**).

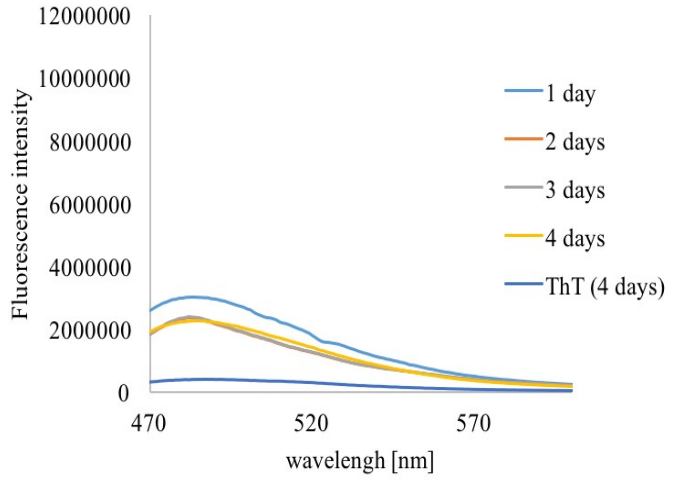

Figure S72. Fluorescence spectra of H-ValGluAlaLeuTyrLeu-OH (**17**).

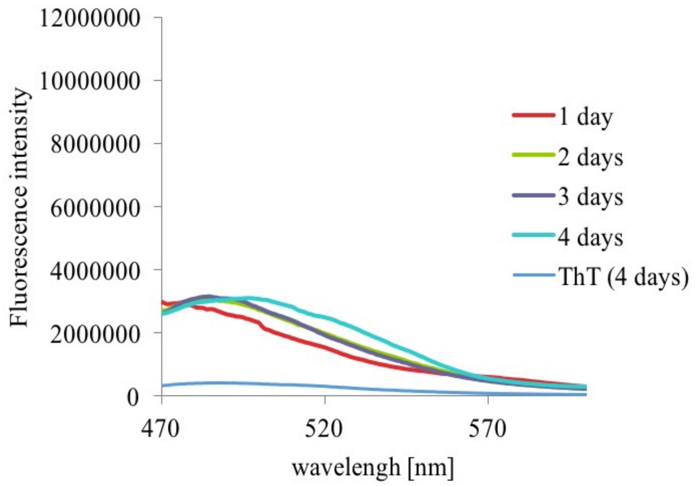

Figure S73. Fluorescence spectra of H-ArgGlyPhePheTyrThr-OH (**18**).

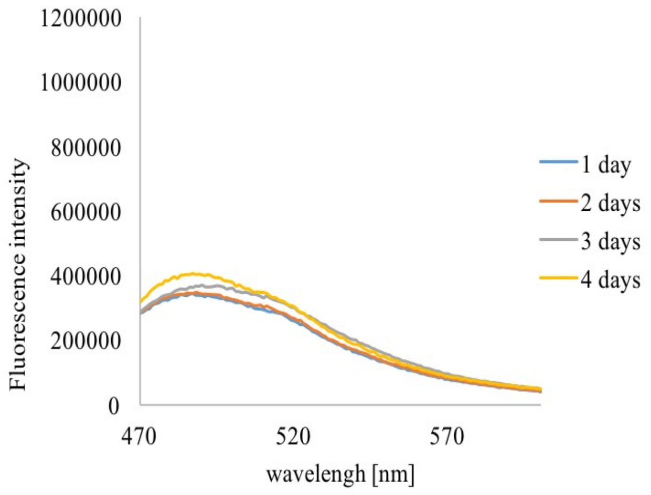

Figure S74. Fluorescence spectra of Thioflavin T.
